# Congenital deficiency reveals critical role of ISG15 in skin homeostasis

**DOI:** 10.1172/JCI141573

**Published:** 2022-02-01

**Authors:** Muhammad Nasir Hayat Malik, Syed Fakhar-ul-Hassnain Waqas, Jana Zeitvogel, Jingyuan Cheng, Robert Geffers, Zeinab Abu-Elbaha Gouda, Ahmed Mahrous Elsaman, Ahmed R. Radwan, Matthias Schefzyk, Peter Braubach, Bernd Auber, Ruth Olmer, Mathias Müsken, Lennart M. Roesner, Gisa Gerold, Sven Schuchardt, Sylvia Merkert, Ulrich Martin, Felix Meissner, Thomas Werfel, Frank Pessler

**Affiliations:** 1 Research Group Biomarkers for Infectious Diseases, TWINCORE, Centre for Experimental and Clinical Infection Research, Hannover, Germany.; 2 Department Dermatology and Allergy, Hannover Medical School, Hannover, Germany.; 3 Experimental Systems Immunology, Max Planck Institute of Biochemistry, Martinsried, Germany.; 4 Genome Analytics, Helmholtz Centre for Infection Research, Braunschweig, Germany.; 5 Department of Dermatology, Venereology and Andrology, Faculty of Medicine, and; 6 Department of Rheumatology and Rehabilitation, Sohag University, Sohag, Egypt.; 7 Institute for Pathology,; 8 Institute for Human Genetics,; 9 Leibniz Research Laboratories for Biotechnology and Artificial Organs (LEBAO), Department of Cardiothoracic, Transplantation and Vascular Surgery (HTTG), and; 10 REBIRTH–Research Center for Translational Regenerative Medicine, Biomedical Research in Endstage and Obstructive Lung Disease Hannover (BREATH), German Center for Lung Research (DZL), Hannover Medical School, Hannover, Germany.; 11 Central Facility for Microscopy, Helmholtz Centre for Infection Research, Braunschweig, Germany.; 12 Institute for Experimental Virology, TWINCORE, Centre for Experimental and Clinical Infection Research, Hannover, Germany.; 13 Department of Bio- and Environmental Analytics, Fraunhofer Institute for Toxicology and Experimental Medicine, Hannover, Germany.; 14 Institute of Innate Immunity, Department of Systems Immunology and Proteomics, Medical Faculty, University of Bonn, Bonn, Germany; 15 Centre for Individualised Infection Medicine, Hannover, Germany.; 16 Helmholtz Centre for Infection Research, Braunschweig, Germany.

**Keywords:** Autoimmunity, Collagens, Monogenic diseases, Skin

## Abstract

Ulcerating skin lesions are manifestations of human ISG15 deficiency, a type I interferonopathy. However, chronic inflammation may not be their exclusive cause. We describe two siblings with recurrent skin ulcers that healed with scar formation upon corticosteroid treatment. Both had a homozygous nonsense mutation in the *ISG15* gene, leading to unstable ISG15 protein lacking the functional domain. We characterized *ISG15^–/–^* dermal fibroblasts, HaCaT keratinocytes, and human induced pluripotent stem cell–derived vascular endothelial cells. ISG15-deficient cells exhibited the expected hyperinflammatory phenotype, but also dysregulated expression of molecules critical for connective tissue and epidermis integrity, including reduced collagens and adhesion molecules, but increased matrix metalloproteinases. *ISG15^–/–^* fibroblasts exhibited elevated ROS levels and reduced ROS scavenger expression. As opposed to hyperinflammation, defective collagen and integrin synthesis was not rescued by conjugation-deficient ISG15. Cell migration was retarded in *ISG15^–/–^* fibroblasts and HaCaT keratinocytes, but normalized under ruxolitinib treatment. Desmosome density was reduced in an *ISG15^–/–^* 3D epidermis model. Additionally, there were loose architecture and reduced collagen and desmoglein expression, which could be reversed by treatment with ruxolitinib/doxycycline/TGF-β1. These results reveal critical roles of ISG15 in maintaining cell migration and epidermis and connective tissue homeostasis, whereby the latter likely requires its conjugation to yet unidentified targets.

## Graphical abstract

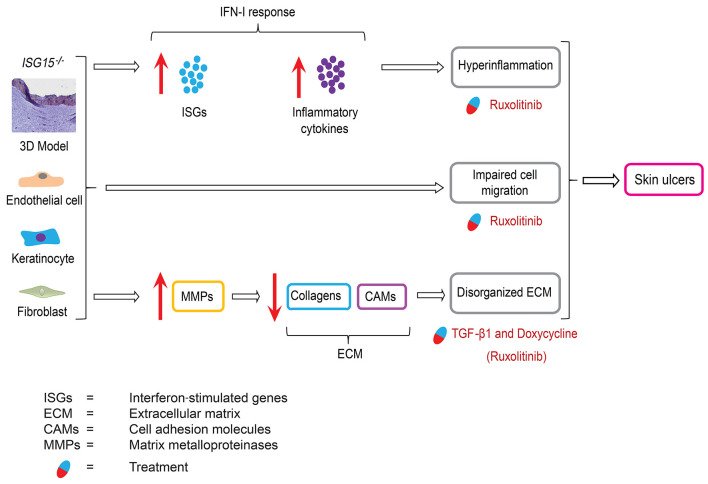

## Introduction

Monogenic type I interferonopathies are a heterogeneous group of autoinflammatory and autoimmune disorders characterized by persistently elevated levels of type I interferons (IFN-I) (1–3). The underlying molecular mechanisms are diverse and include abnormal accumulation of endogenous nucleic acids as in Aicardi-Goutières syndrome and familial chilblain lupus (4), enhanced sensitization of IFN-I receptors (IFNARs) as in STING-associated vasculopathy with onset in infancy (SAVI; ref. 5), enhanced IFN-I gene transcription due to gain of function of STAT2 (6, 7), and defective negative regulation of IFN-I due to loss of IFN-stimulated gene 15 (*ISG15*; ref. 8) or ubiquitin-specific peptidase 18 (USP18; ref. 9) (reviewed in refs. 3, 10). ISG15 deficiency was the first example of a type I interferonopathy due to an inborn defect in the negative-feedback loop involving ISG15 and its downstream effector USP18, where absence of ISG15 protein leads to a secondary loss of USP18 and its ability to downregulate signaling through the IFNAR (8, 11). ISG15 deficiency originally came to clinical attention as one etiology of Mendelian susceptibility to mycobacterial disease (MSMD), where loss of free ISG15 leads to compromised induction of IFN-γ (12). Subsequent autoinflammation-related clinical findings of *ISG15* deficiency were manifestations of systemic inflammation, cerebral calcifications, and epileptic seizures, all of which are consistent with chronic hyperinflammation, originating, for instance, from innate immune cells such as macrophages and, in the case of CNS inflammation, their counterpart in CNS, microglia (11). However, skin lesions were recently documented in several patients (13, 14). This was initially not too surprising since vasculitic skin lesions are common in the monogenic type I interferonopathies SAVI and familial chilblain lupus (3, 15), and also in polygenically determined autoimmune diseases with IFN signatures such as juvenile systemic lupus erythematosus (16). However, histological changes in skin lesions in those cases of ISG15 deficiency in which tissue biopsies were available did not show bona fide vasculitis (13).

*ISG15* is one of the most highly upregulated genes in IFN-I responses. Its product is a versatile protein that engages in a multitude of protein-protein interactions (“ISGylations”) and, in humans, maintains an ISGylation-independent negative-feedback loop that leads to downregulation of IFN-signaling by stabilizing USP18 (11). In addition, accumulating evidence has revealed that *ISG15* can also regulate a variety of processes whose activity does not strictly depend on the IFN response, notably apoptosis (17), autophagy (18), cell respiration (19), and synthesis of reactive oxygen species (ROS) (19). However, an involvement in connective tissue and/or skin homeostasis has not been described. We have investigated two siblings with unexplained recurring skin ulcerations that healed with scar formation under corticosteroid treatment and, unexpectedly, identified a homozygous nonsense mutation of *ISG15* as the most plausible etiology in both cases. We have, therefore, investigated potential roles of *ISG15* in homeostasis of connective tissue and skin epithelium. We find that *ISG15* is required for physiological expression of major constituents of connective tissue such as collagens, for normal cell migration, and for orderly formation of desmosomes and an epidermis-like structure in a 3D model. These findings (a) add cell migration, connective tissue homeostasis, and epidermis formation to the list of *ISG15* functions that go beyond regulating IFN signaling; (b) provide further evidence that *ISG15* deficiency should be considered in the differential diagnosis of ulcerating skin lesions of childhood when an infectious etiology cannot be readily identified; and (c) suggest potential treatments for this rare disorder.

## Results

### Clinical case description.

**Figure 1:**
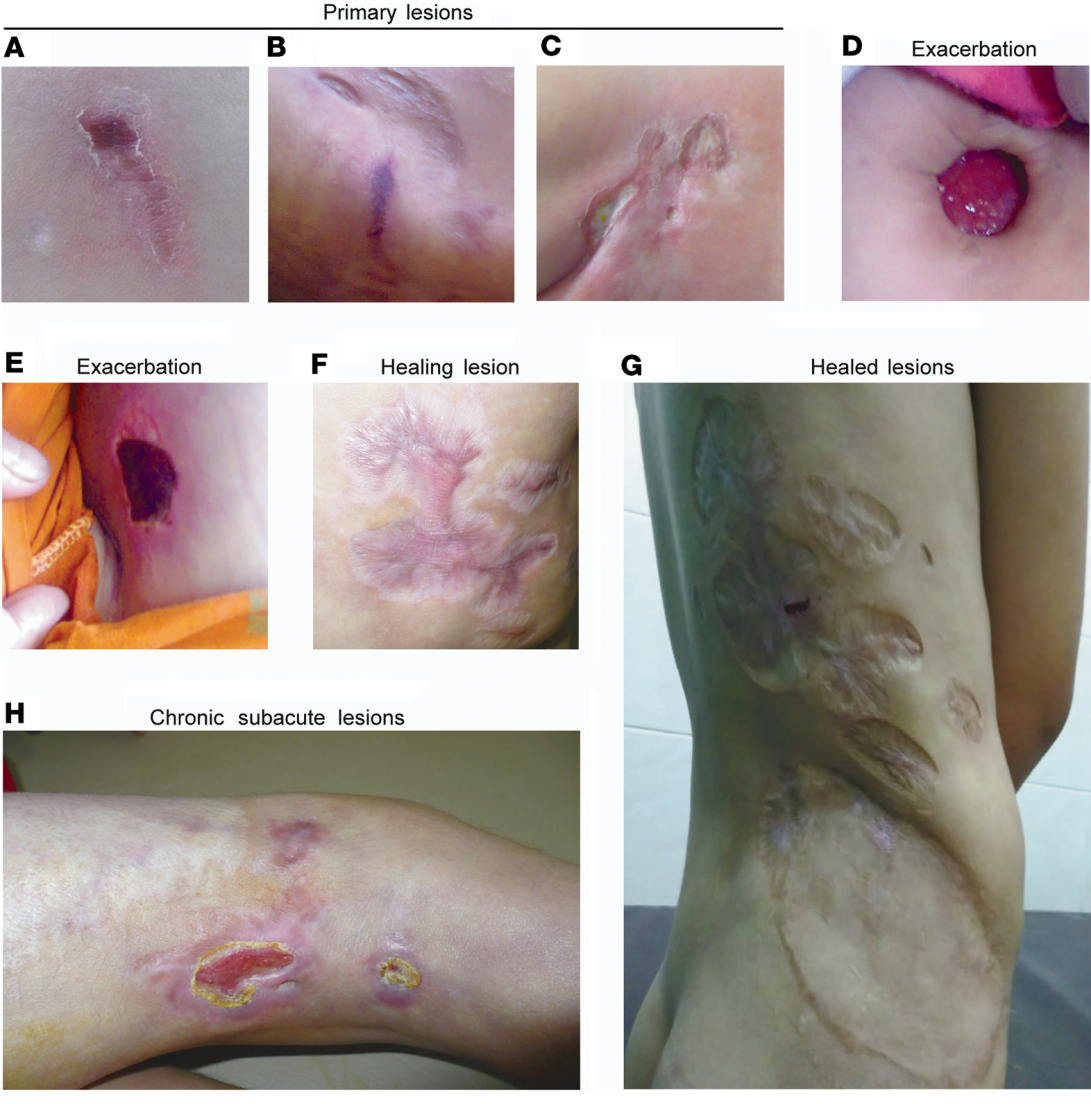
Macroscopic appearance of skin lesions in different clinical stages. (**A** and **B**) Violaceous, nonblanching primary lesions with the characteristic appearance of a superficial vasculitis. (**C**) Superficially ulcerating primary lesion. (**D** and **E**) Deep necrotizing ulcerations during exacerbation. (**F**) Scariform lesions in an intermediate stage of healing. (**G**) Extensive scar formation seen in completely healed lesions in P1 during remission. (**H**) Chronic subacute lesion on right knee joint. **B** and **D**–**H** show lesions from P1, **A** and **C** from P2.

**Figure 2:**
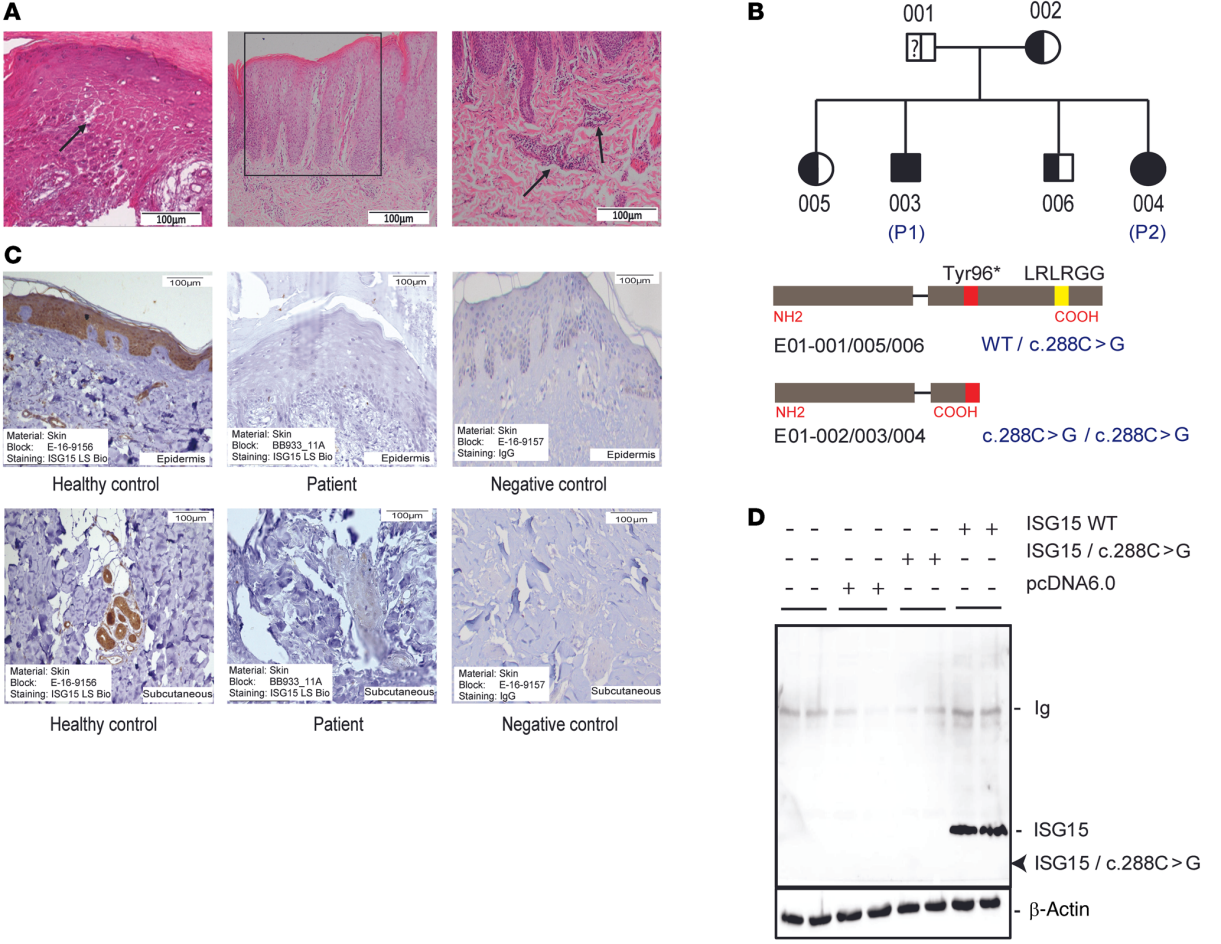
Histological and genetic features. (**A**) Selected histological findings (P1). Left: H&E stains from a biopsy of an active skin lesion demonstrating intercellular edema (spongiosis; arrow) and parakeratosis reminiscent of an eczematous lesion. Middle and right: H&E stains from a biopsy of a chronic lesion demonstrating psoriasiform epidermal hyperplasia (box) and perivascular infiltration (arrows). Scale bars: 100 μm. (**B**) Results of whole exome sequencing: The index cases are homozygous for a nonsense mutation c.288C>G, which is predicted to lead to a truncated ISG15 protein missing the active domain. Sequencing depth of the WT and/or mutant allele in each family member is shown in Supplemental Table 2. (**C**) IHC staining for ISG15 protein with a primary antibody directed against the C-terminus (aa 136–165). There is strong expression in epidermis and in dermal blood vessels in control tissue, which is absent in the index case (chromogen: DAB, brown). Nonspecific rabbit IgG was used as negative control. Scale bars: 100 μm. (**D**) The truncated ISG15 protein resulting from the c.288C>G mutation is unstable. *ISG15^–/–^* fibroblasts were transfected with plasmids expressing WT or c.288C>G ISG15 coding sequences and tested for expression of ISG15 by immunoblot using an antibody directed against amino acids 1–150, thus also recognizing the NH_2_-terminus. A band of the expected migration of the truncated protein is not seen (arrow).

A 9-month-old boy from Upper Egypt (P1), the son of consanguineous parents (second cousins), developed a violaceous and erythematous tender plaque in the inguinal region that did not blanch with pressure and had the characteristic “vasculitic hue” (images of lesions from both patients at different clinical stages are shown in Figure 1). The lesion subsequently increased in size and ulcerated, also revealing intense erythema of the exposed tissue. The ulcer was treated with a skin graft, which healed well without recurrence. Ulcerations recurred after 3 years, now also involving the perianal region; owing to their similarity to ulcerating lesions of cutaneous tuberculosis (TB) (20) and the high local incidence of TB, the patient was treated with a full course of anti-TB drugs, and the lesions eventually healed with scar formation. To rule out polyarteritis nodosa, a head CT was performed on P1 at age 5 years and revealed CNS calcifications. At age 7, similar lesions recurred in the same location and also spread to other body parts, including trunk and knee joints. At this age he also developed fevers and painful knee swelling, but knee MRI did not reveal evidence of arthritis. The lesions progressed despite a second course of anti-TB treatment. A lesional skin biopsy did not reveal granulomas, and a stain for acid-fast bacilli was therefore not done, but PCR for *Mycobacterium* *tuberculosis* was negative, as were tests for other regionally relevant infectious etiologies. The histological picture was interpreted as being suggestive of an inflammatory process, and subsequent chronic treatment with corticosteroids led to a partial remission. Five years after the birth of P1, the parents had a daughter (P2), who developed identical-appearing lesions (also at age 9 months), raising the suspicion of a genetic etiology. She also experienced partial remission on corticosteroid treatment, but disease flared in both siblings when corticosteroids were tapered. Methotrexate was subsequently added, which appeared to aid in lesion healing and to reduce the frequency of flares in both cases. However, P1 intermittently required more aggressive treatments during flares with mycophenolate mofetil, cyclophosphamide, and long-acting penicillin, and P2 was additionally treated with azithromycin (1 course per month) as needed. Notably, all healed lesions left cosmetically disfiguring scars. At the most recent assessment at age 14 (P1) and 9 (P2), P1 had experienced a last flare 6 months before, which remitted spontaneously, and at time of writing he remained in remission off treatment. P2 still required treatment for recurrences at time of writing. Past exposure to and current infection with *M.* *tuberculosis* were subsequently ruled out in both patients by negative skin test and IFN-γ release assay. Bacille Calmette-Guérin vaccination, administered in the first year of life, did not lead to overt complications in either patient (although targeted exams for regional lymphadenopathy were not done), and the injection sites healed without scar formation. Both patients have demonstrated grossly normal psychoneurological development and no history of seizures. Findings of brain MRI, performed in the patients at age 12 and 7 years, respectively, were within normal limits. Autoantibody screening in peripheral blood was repeated for the boy at that age, since autoimmunity may develop later in the course of ISG15 deficiency (8). Low-titer anti-nuclear antibodies (1:40, homogeneous pattern) were detected in the boy (at 12 years) but not in the girl (at age 7 years), and both were negative for anti-neutrophil cytoplasmic antibodies (Supplemental Table 1; supplemental material available online with this article; https://doi.org/10.1172/JCI141573DS1). The boy also had elevated erythrocyte sedimentation rate (106 mm after 2 hours) and C-reactive protein levels (24 mg/L). Reevaluation of the H&E-stained sections of the skin biopsies (Figure 2A) revealed superficial lymphohistiocytic spongiotic dermatitis in both patients. In the boy (P1), histological changes comprised neutrophilic infiltrates around small ectatic and hyperemic blood vessels, thin suprapapillary plates, central parakeratosis (retention of nuclei in stratum corneum suggesting defective keratinocyte differentiation), thinning of the stratum granulosum, and psoriasiform acanthosis. In the girl (P2), erosions, focal epidermal necrosis, and serum with massive bacterial accumulations were seen, which was consistent with a superinfected eczematous lesion. Bona fide vasculitis was not seen in either biopsy.

Whole exome sequencing (WES) of peripheral blood DNA revealed that P1 and P2 were homozygous for a nonsense mutation in the *ISG15* gene (c.288C>G), which resulted in a premature stop codon at the Tyr96 position (p.Tyr96*), whereas the mother and the unaffected siblings were heterozygous for this mutation (Figure 2B). Notably, the mutant allele was not detected in the father. Analysis of short tandem repeats (Supplemental Table 3) and X chromosome haplotypes (Supplemental Table 4) verified paternity and correctness of the pedigree including assignment of the WES data files, suggesting a spontaneous germline mutation, germline mosaic, or deletion of the variant allele in peripheral blood cells in the father. The index cases were predicted to express a truncated nonfunctional ISG15 protein lacking the C-terminal functional domain. Indeed, immunohistochemistry (IHC) with an antibody directed against the C-terminus of ISG15 demonstrated the absence of specific staining in the lesional skin biopsy from P1, whereas strong specific staining in epidermis and in and around blood vessels was seen in control skin (Figure 2C). To test whether overall stability of the truncated form was affected, we transiently transfected *ISG15^–/–^* fibroblasts with plasmids expressing *ISG15* WT or *ISG15* mutant (c.288C>G) and analyzed cell lysates for ISG15 protein expression by immunoblot using an antibody also recognizing the N-terminus. A band corresponding to the predicted truncated fragment was not detected, suggesting instability of the truncated polypeptide or nonsense-mediated decay of the mRNA (Figure 2D).

Considering the severity of the patients’ skin phenotypes, we searched for homozygous non-synonymous rare variants that were present in the index patients but not the unaffected siblings and the parents. This analysis identified 12 single-nucleotide variants in 8 genes, 5 of which had been associated with skin development, integrity, or inflammation (Supplemental Table 5). However, no information regarding functional alterations or disease associations could be found in the NCBI’s dbSNP database (www.ncbi.nlm.nih.gov/snp/) about these variants. Realizing the limitations of using WES data for a bona fide copy number variation analysis, we performed a gene deletion analysis with the kindred using the HMZDelFinder tool (https://github.com/casanova-lab/HMZDelFinder_opt), which was recently improved for this purpose (21). This analysis did not reveal any deletions in P2, whereas unique deletions in the *KRTAP9* gene (which plays roles in formation of hair fiber structure) and the *GSTT1* gene (encoding an enzyme important for intracellular redox homeostasis) were found in P1 (Supplemental Table 6). However, the latter deletion is a common variant found in more than a third of the general population.

### IFN-I signature in ISG15^–/–^ fibroblasts.

To test for hyperinflammation in a cell type widely found in skin and connective tissue, we stimulated WT and *ISG15^–/–^* dermal fibroblasts with IFN-α2b. Further tissue samples or cells from the patients were not available because the parents refused for sociocultural reasons. We therefore used previously published human telomerase reverse transcriptase–immortalized (hTERT-immortalized) dermal fibroblast cell lines from a different patient with ISG15 deficiency and a healthy control (22). Consistent with our previous findings (13), transcription of ISGs (*IFIT1*, *OAS1*, and *MX1*) was increased in the *ISG15^–/–^* cells after IFN-α stimulation. In addition, *CXCL10* (a major IFN-induced proinflammatory chemokine) mRNA was also upregulated in *ISG15^–/–^* fibroblasts, further indicating a hyperinflammatory state (Supplemental Figure 1). These results suggested that these cells possessed the expected phenotype of ISG15 deficiency and could be used to search for pathways involved in the pathogenesis of skin lesions in ISG15 deficiency.

### Proteome analysis reveals oxidative stress and collagen dysregulation in ISG15^–/–^ fibroblasts.

**Figure 3:**
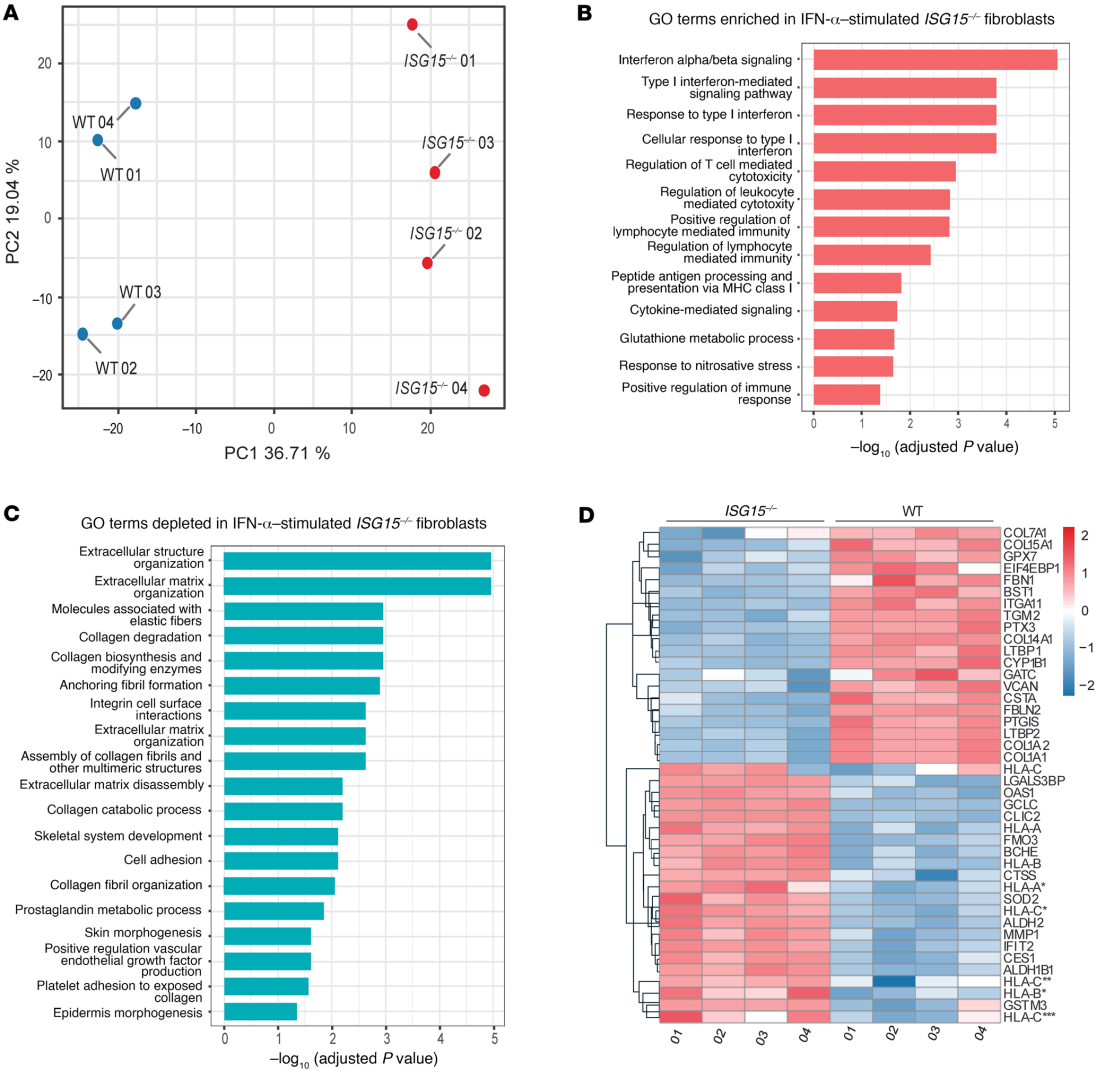
Proteomic analysis reveals major dysregulation in connective tissue homeostasis in *ISG15^–/–^* fibroblasts after IFN-α stimulation. Immortalized dermal fibroblasts carrying either the native ISG15 locus (WT) or a naturally occurring loss-of-function mutation (*ISG15^–/–^*) were stimulated with IFN-α (1000 IU/mL) for 24 hours and subjected to global proteome analysis (*n* = 4). (**A**) Principal component analysis demonstrating clear separation between WT and *ISG15^–/–^* cells based on their protein expression patterns. (**B** and **C**) Enrichment analysis based on the proteome data used as input for **A**. GO terms relating to IFN responses, other inflammatory processes, and stress responses are enriched in *ISG15^–/–^* cells (**B**), whereas GO terms relating to epidermal and connective tissue/extracellular matrix homeostasis are depleted in *ISG15^–/–^* cells (**C**). *X* axes show Benjamini-Hochberg adjusted *P* values (–log_10_) for enrichment/depletion; only GO terms with adjusted *P* less than 0.05 are shown. (**D**) Hierarchical clustering of differentially abundant (adjusted *P* < 0.05) proteins showing upregulation of proinflammatory proteins, HLA molecules, and MMP1 but downregulation of collagen constituents and ROS scavengers in *ISG15^–/–^* cells.

To identify pathophysiological mechanisms, we then performed a whole-proteome analysis of IFN-α–primed WT and *ISG15^–/–^* fibroblasts. In a principal component analysis, protein abundance profiles of IFN-α–primed WT and *ISG15^–/–^* cells were distinct (Figure 3A). Gene Ontology (GO) enrichment analysis revealed the expected enrichment (upregulation) of IFN-I– and cytokine-mediated signaling pathways in *ISG15^–/–^* cells, but also induction of glutathione metabolism (indicating oxidative stress) and nitrosative stress (Figure 3B). Depleted GO terms related to various aspects of connective tissue integrity and extracellular matrix, including collagen metabolism, cell-cell adhesion, integrin interactions, and extracellular matrix organization pathways (Figure 3C). Consistent with the latter, GO terms related to skin and epidermis morphogenesis were also depleted in the *ISG15^–/–^* cells. Inspection of the underlying protein abundances (Figure 3D) confirmed the expected upregulation of ISGs, of which OAS1 and IFIT2 were most significantly increased. Mitochondrial aldehyde dehydrogenase 2 (ALDH2) and superoxide dismutase 2 (SOD2), which are induced during oxidative stress (23), and proteins reflecting nitrosative stress were also upregulated in these *ISG15^–/–^* fibroblasts. We then investigated expression of matrix metalloproteinases (MMPs) and collagens, as both play key roles in skin integrity and wound healing (24). As expected, there was broad repression of collagens (COL1A1, COL5A2, COL7A1, COL12A1, COL14A1, and COL15A1) in the *ISG15^–/–^* cells, but also a prominent reduction in cell adhesion molecules (e.g., ITGA11 and TGM2), and — interestingly — increased expression of MMP1 (Figure 3D). We also noted reduced expression of latent TGF-β–binding proteins (LTBPs) 1 and 2, which augment stability and secretion of TGF-β1 (25, 26). Together, these findings suggested that dysregulation of collagen homeostasis and expression of adhesion molecules might act in concert with oxidative stress and hyperinflammation in the pathogenesis of skin ulcerations in ISG15 deficiency.

### Increased ROS and reduced AKT phosphorylation modulate MMP1 and collagen expression in ISG15^–/–^ dermal fibroblasts.

**Figure 4:**
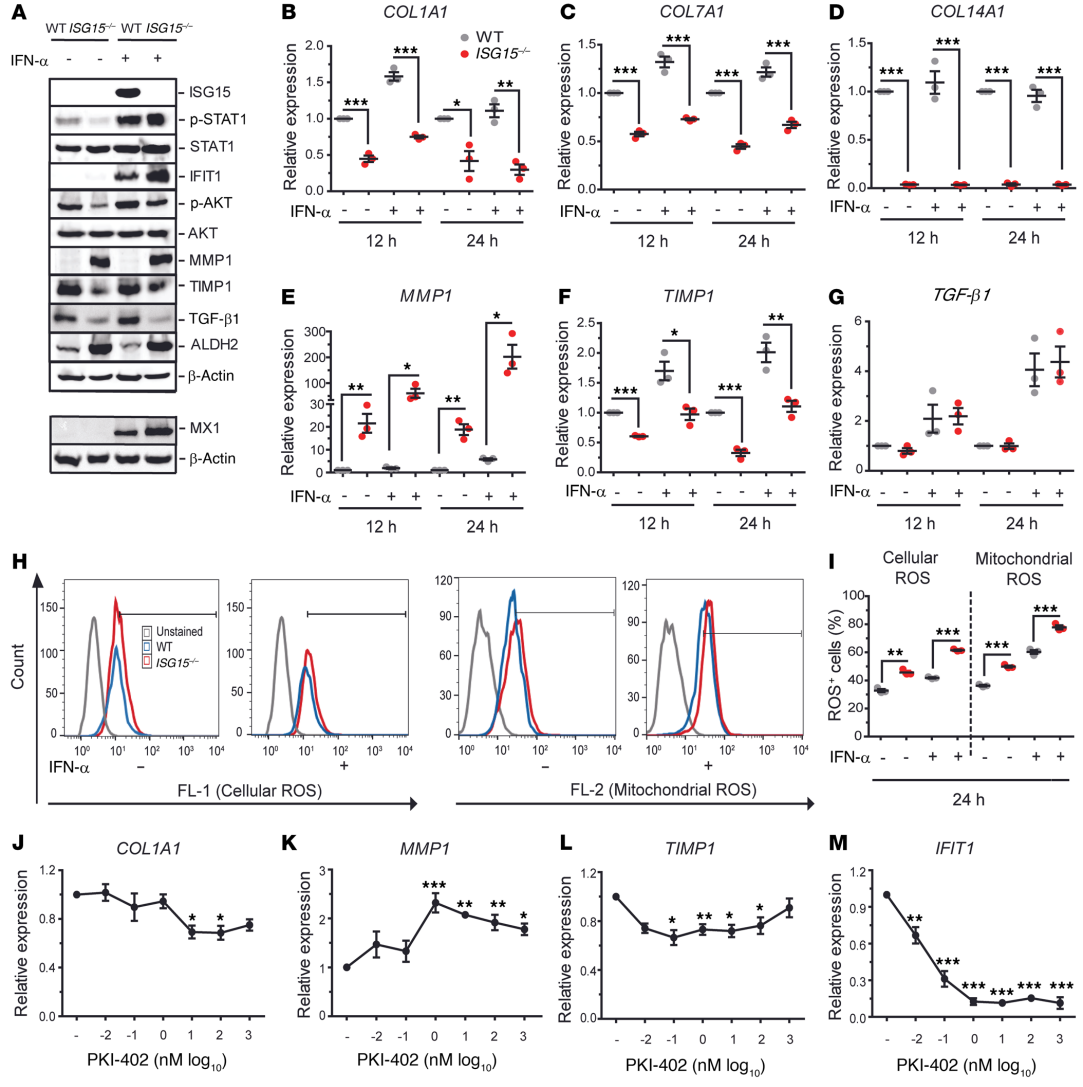
Validation of dysregulated collagen homeostasis, increased type I IFN activity, and increased oxidative stress in *ISG15^–/–^* fibroblasts. WT or *ISG15^–/–^* immortalized fibroblasts were stimulated with IFN-α (1000 IU/mL) for the indicated times and analyzed by RT-qPCR, immunoblot, and flow cytometry. (**A**) Immunoblots showing increased IFIT1, MX1, MMP1, and ALDH2 but decreased phosphorylated AKT and near-absent TGF-β1 in *ISG15^–/–^* cells. The MX1 and corresponding β-actin blots were obtained from a separate experiment. (**B**–**G**) RT-qPCR analysis revealing decreased expression of collagens (**B**–**D**), increased expression of *MMP1* but decreased *TIMP1* expression in *ISG15^–/–^* cells, but no apparent change in *TGF-β1* mRNA (**E**–**G**). ΔΔCt analysis, with expression in unstimulated WT cells arbitrarily assigned the reference value of 1. (**H** and **I**) Determination of mitochondrial and cellular ROS (mtROS, cROS) by flow cytometry. Total numbers and percentages of mtROS- and cROS-positive cells are higher among the *ISG15^–/–^* cells. (**J**–**M**) Addition of the PI3K inhibitor PKI-402 phenocopies reduced *COL1A1*, *TIMP1*, and *IFIT1* (**J**, **L**, and **M**) and increased *MMP1* (**K**) expression in human fibroblasts. RT-qPCR, ΔΔCt analysis; reference = expression in untreated cells. *n* = 3, ± SEM; *P < 0.05, **P < 0.01, ***P < 0.001 (**B**–**I**, Student’s *t* test; **J**–**M**, 1-way ANOVA with Dunnett’s post hoc test).

To validate the proteomic findings, we stimulated WT and *ISG15^–/–^* fibroblasts with IFN-α2 and measured expression of selected targets by reverse transcription quantitative PCR (RT-qPCR) and immunoblot. There was marked upregulation of IFIT1 and MX1 proteins, and a modest increase (IFN-α–stimulated *ISG15^–/–^* vs. WT cells: 47,937 vs. 37,400 normalized densitometry units) in phosphorylated STAT1 (p-STAT1; Figure 4A). We further noted downregulation of *COL1A1*, *COL7A1*, and *COL14A1* mRNA (Figure 4, B–D) and upregulation of *MMP1* mRNA (Figure 4E) but, interestingly, downregulation of tissue inhibitor of matrix metalloproteinase 1 (*TIMP1*; Figure 4F) in the *ISG15^–/–^* fibroblasts. In contrast to the decreased TGF-β1 expression observed by proteomics, there was no difference in *TGF-β1* mRNA expression (Figure 4G). However, there was a remarkable reduction at the protein level (Figure 4A), suggesting a posttranslational modification likely owing to the reduced LTBP levels detected by proteomics.

To verify the causal relationship between reduced TGF-β1 levels and aberrant *MMP1* and *COL1A1* expression, we used siRNA to knock down *TGF-β1* mRNA in WT and *ISG15^–/–^* cells, and *ISG15^–/–^* cells stably transduced with vectors expressing either WT ISG15 protein or a conjugation-deficient ISG15 mutant (Supplemental Figure 2). An approximately 50% knockdown was achieved, and a corresponding upregulation of *MMP1* and downregulation of *COL1A1* mRNA were indeed observed in the WT and in the WT-transduced *ISG15^–/–^* cells. Interestingly, similar changes were observed in the *ISG15^–/–^* cells, possibly due to abrogation of the remaining TGF-β1 expression seen in these cells in Figure 4A. Knockdown in the cells expressing the conjugation-deficient ISG15 variant led to a paradoxical increase of *COL1A1* expression of unknown significance (Supplemental Figure 2K).

The immunoblot also confirmed higher levels of mitochondrial ALDH2 in *ISG15^–/–^* cells, indicating mitochondrial stress (Figure 4A). Flow cytometry revealed enhancement of cellular and mitochondrial ROS in *ISG15^–/–^* fibroblasts (Figure 4, H and I). It was recently shown that ROS can inhibit PI3K (27), which can regulate MMP1 expression (27); we therefore measured AKT phosphorylation and indeed observed reduced p-AKT in the *ISG15^–/–^* cells (Figure 4A).

### PI3K inhibition phenocopies MMP1 and collagen dysregulation due to ISG15 deficiency in fibroblasts.

Considering the decreased AKT phosphorylation in *ISG15^–/–^* fibroblasts, which could be associated with enhanced *MMP1* and reduced *TIMP1* levels, we applied the PI3K inhibitor PKI-402 to WT fibroblasts. Short-term treatment (3 hours) with PKI-402 sufficed to reduce *COL1A1* (Figure 4J), induce *MMP1* (Figure 4K), and reduce *TIMP1* (Figure 4L) mRNA levels. In addition, we observed a strong reduction in *IFIT1* expression (Figure 4M), which is a known effect of PI3K inhibitors (28). Similar results were obtained by longer treatment (24 hours), except that suppression of *TIMP1* expression was no longer observed at this time point (Supplemental Figure 3). In order to test whether the action of PKI-402 required the presence of active ISG15 and correlated with reduced AKT phosphorylation, we then applied the inhibitor to both WT and *ISG15^–/–^* cells (Supplemental Figure 4). PKI-402 markedly reduced AKT phosphorylation in both WT and *ISG15^–/–^* cells, indicating that it can fully function in *ISG15^–/–^* cells. In addition, even though the changes were less pronounced, it altered expression of *MMP1* and *COL1A1* in the same direction in WT and *ISG15^–/–^* cells. Taken together, these data indicate that the observed dysregulation of *MMP1* and *COL1A1* expression in *ISG15^–/–^* fibroblasts is partially due to inhibition of AKT signaling and that their expression is additionally regulated by ISG15-independent AKT signaling.

### Hyperinflammation and collagen dysregulation in ISG15-deficient vascular endothelial cells derived from human induced pluripotent stem cells.

**Figure 5:**
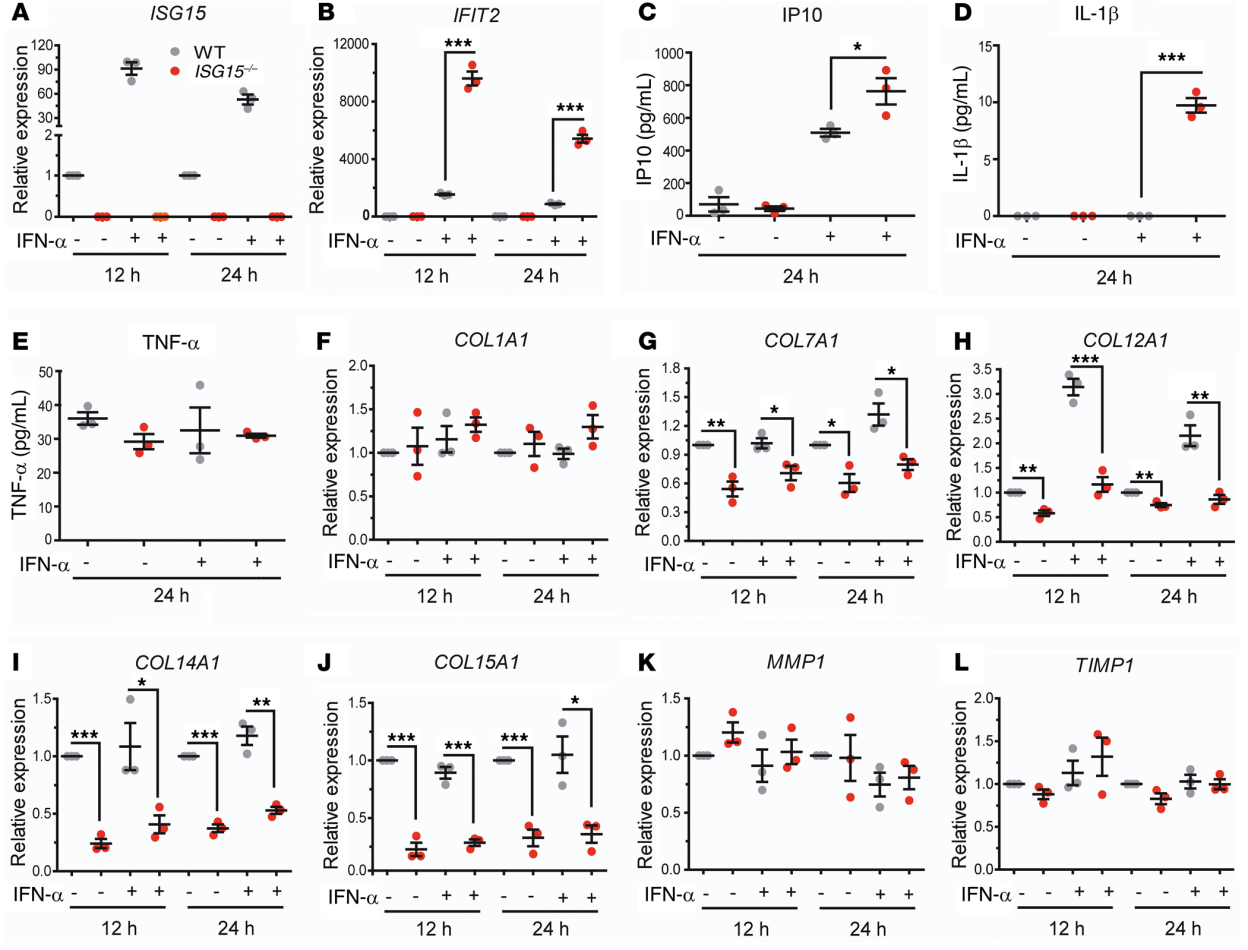
Hyperinflammation and decreased collagen expression in *ISG15^–/–^* vascular endothelial cells. Human iPSCs carrying the WT *ISG15* locus or a CRISPR/Cas9-mediated targeted deletion were differentiated into CD31-positive vascular ECs and stimulated with IFN-α (1000 IU/mL) for 12 or 24 hours. (**A**) Absence of *ISG15* mRNA in *ISG15^–/–^* ECs. (**B**–**E**) Increase in intracellular *IFIT2* mRNA, and IP10 and IL-1β but not TNF-α protein in culture supernatants. (**F**–**L**) Decreased expression in several collagen mRNAs but not *COL1A1*, *MMP1*, and *TIMP1* in *ISG15^–/–^* ECs. RT-qPCR with ΔΔCt analysis; reference = unstimulated WT cells (**A**, **B**, and **F**–**L**), or enzyme-linked immunoassay (**C**–**E**). *n* = 3, ± SEM; *P < 0.05, **P < 0.01, ***P < 0.001 (Student’s *t* test).

In order to test whether the observed dysregulation in fibroblasts also affected other cell types that play critical roles in skin inflammation and homeostasis, we evaluated the effects of ISG15 deficiency in endothelial cells (ECs) derived from human induced pluripotent stem cells (hiPSCs). hiPSCs were used because they can be differentiated into various nontransformed cell types that have phenotypes close to the corresponding primary cell types. We therefore generated *ISG15^–/–^* hiPSCs with CRISPR/Cas9 and subsequently differentiated them (as well as the isogenic WT clone) into hiPSC-ECs. Absence of *ISG15* mRNA in the knockout cells and induction by IFN-α in the WT cells were verified by RT-qPCR (Figure 5A). The *ISG15^–/–^* cells were hyperinflammatory, as evidenced by markedly higher induction of *IFIT2* mRNA, and higher release of IL-1β and IP10 protein into the supernatant upon treatment with IFN-α (Figure 5, B–D). However, there were no significant changes in *TNFA* and *COL1A1* mRNA levels (Figure 5, E and F). Whereas expression of *COL7A1*, *COL12A1*, *COL14A1*, and *COL15A1* mRNA was markedly lower in the *ISG15^–/–^* ECs both before and after IFN-α stimulation (Figure 5, G–J), *MMP1* and *TIMP1* mRNA expression was similar in WT and *ISG15^–/–^* cells (Figure 5, K and L). Thus, ISG15 deficiency in hiPSC-ECs induced the expected hyperactive IFN-I response and attenuated transcription of collagens, but there were distinct differences in response patterns to IFN-α stimulation in comparison with the fibroblasts.

### Hyperinflammation and collagen dysregulation in ISG15-deficient primary keratinocytes and HaCaT keratinocyte line.

**Figure 6:**
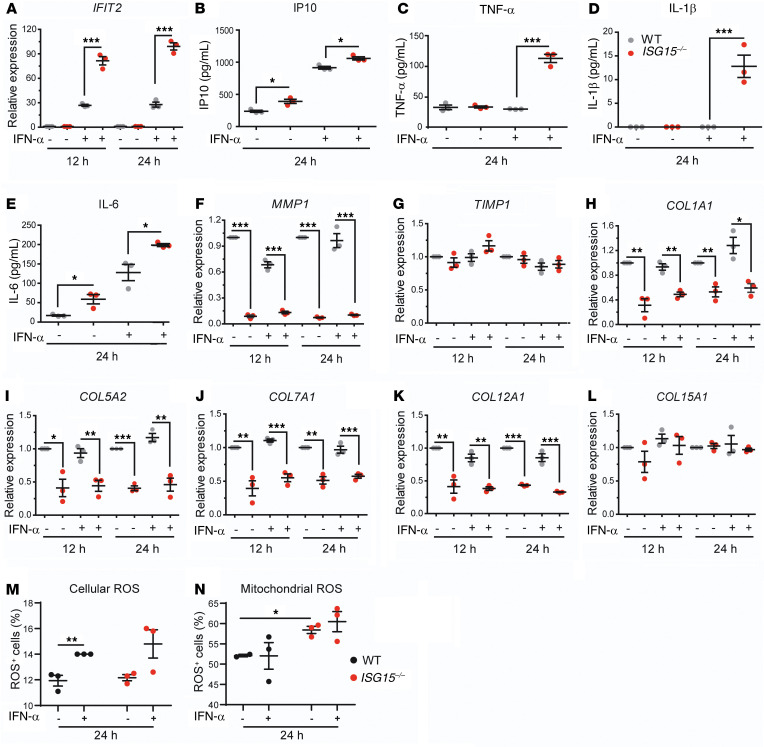
Hyperinflammation and collagen deficiency, but unchanged ROS levels, in *ISG15^–/–^* HaCaT keratinocytes. HaCaT cells were stimulated with IFN-α (1000 IU/mL) for 12 or 24 hours and analyzed by RT-qPCR, enzyme-linked immunoassay, and flow cytometry. (**A**–**E**) Increased *IFIT2* mRNA in cell pellets and IP10, TNF-α, IL-1β, and IL-6 in culture supernatants from *ISG15^–/–^* HaCaT cells. Increased concentrations of TNF-α and IL-1β were seen in *ISG15^–/–^* cells only after IFN-α stimulation. (**F** and **G**) Near absence of *MMP1* mRNA but unchanged expression of *TIMP1* mRNA in *ISG15^–/–^* HaCaT cells. (**H**–**L**) Decreased expression of several collagen mRNAs except *COL15A1* in *ISG15^–/–^* cells. (**M** and **N**) Cellular and mitochondrial ROS. *n* = 3, ± SEM; *P < 0.05, **P < 0.01, ***P < 0.001 (**A**–**L**, Student’s *t* test; **M** and **N**, 1-way ANOVA with Tukey’s post hoc test).

As in fibroblasts and hiPSC-ECs, IFN-α induction of ISGs and inflammatory markers was higher in *ISG15^–/–^* HaCaT cells and primary keratinocytes than in the WT cells (Figure 6, A–E, and Supplemental Figure 5). In contrast to *ISG15^–/–^* fibroblasts, MMP1 levels were actually reduced in *ISG15^–/–^* HaCaT cells (Figure 6F), which is consistent with a prior report that MMP1 expression can differ substantially between fibroblasts and keratinocytes (29). In addition, TIMP1 levels did not differ between WT and *ISG15^–/–^* HaCaT cells (Figure 6G). Nonetheless, collagen transcripts were downregulated in a manner similar to that seen in the other cell types in *ISG15^–/–^* HaCaT cells (Figure 6, H–L) and primary keratinocytes (Supplemental Figure 5). Another difference from fibroblasts was that IFN-α stimulation did not appreciably increase ROS levels in either genotype (Figure 6, M and N). Moreover, after transcript profiling of HaCaT cells with microarrays, GO enrichment analysis revealed decreased cell-cell adhesion and epidermis development in unstimulated *ISG15^–/–^* cells as compared with WT cells (Supplemental Figure 6, A and B). As expected, IFN-α stimulation led to enrichment of pathways relating to inflammatory responses and leukocyte migration in the *ISG15^–/–^* cells compared with the WT cells; moreover, regulation of response to wounding was also among the enriched GO terms; most depleted pathways related to cell differentiation and organ development (Supplemental Figure 6C). Specifically, *ISG15^–/–^* HaCaT cells supported enhanced expression of inflammatory cytokines (e.g., *IL1B*, *CXCL8*, and *IL1A*) and ISGs (e.g., *IFIT1* and *OAS1*), but attenuated expression of the desmosome constituent desmoglein 4 (*DSG4*), cell adhesion molecules such as cadherins (*CDH2*, *CDH8*, and *CDH13*), integrins (e.g., *ITGA4* and *ITGAE*), and TGF-β–induced factor homeobox 2 (*TGIF2*) (Supplemental Figure 6D). TGIF2 can repress transcription of TGF-β1–responsive genes, particularly *SMAD3*, by recruiting histone deacetylases (30). Thus, as in fibroblasts, collagen dysregulation in *ISG15^–/–^* HaCaT cells could also be due to reduced transcription of TGF-β1–responsive genes. Taken together, these results suggest that keratinocytes contribute to skin pathology in ISG15 deficiency not alone by hyperinflammation but also through dysregulation of collagen homeostasis, dysregulated cell differentiation, and weakening of cell-cell interactions.

### Disorganized epidermis due to reduced desmosomes and cell adhesion molecules in an ISG15^–/–^ 3D epidermis model.

**Figure 7:**
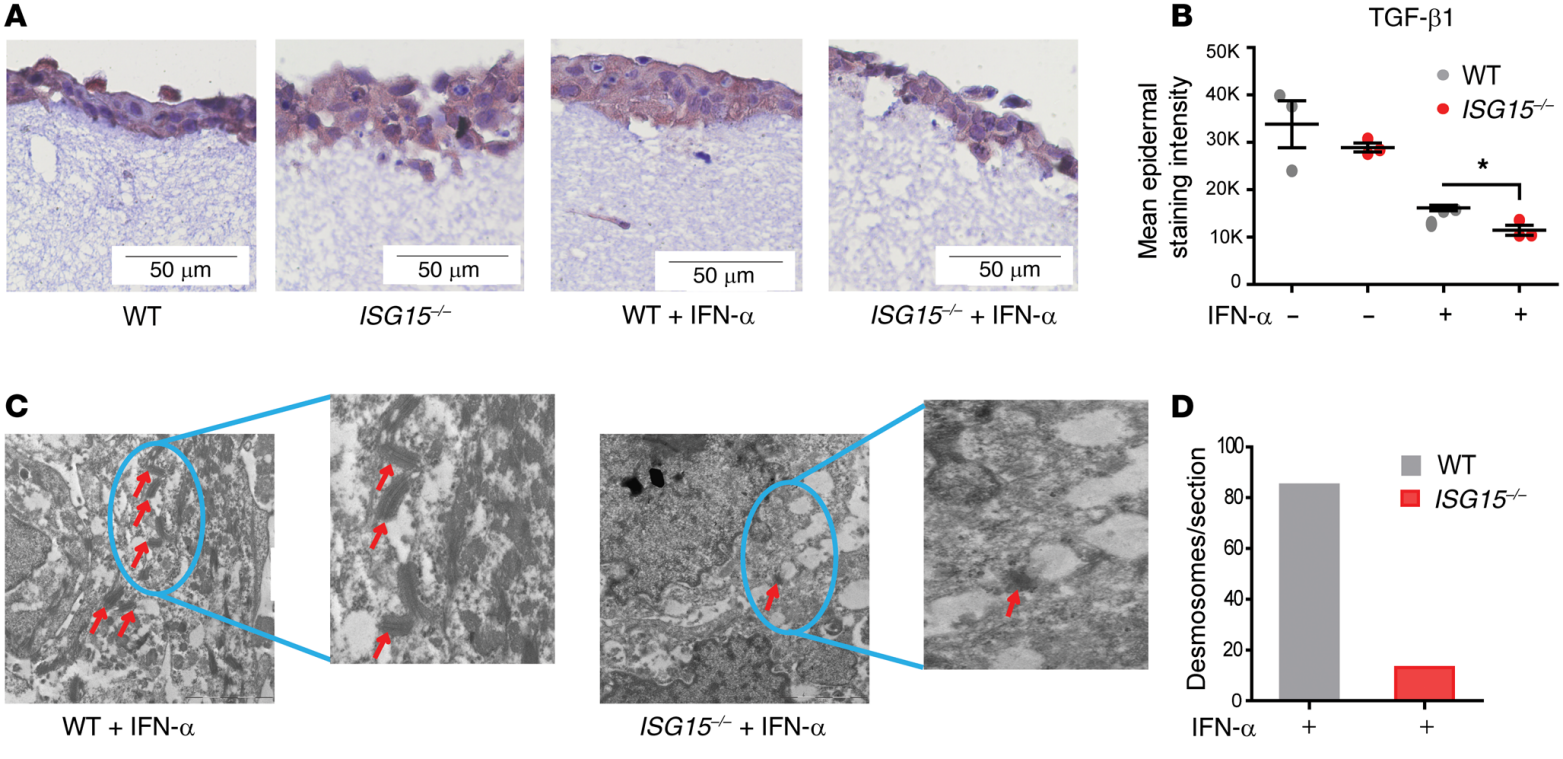
ISG15 deficiency causes histological disorganization and loss of desmosomes in a 3D epidermis model. An epidermis-like structure was formed using coculture of HaCaT cells and immortalized dermal fibroblasts (both WT or both *ISG15^–/–^*) on a collagen matrix and analyzed by light and electron microscopy, RT-qPCR (ΔΔCt method, using unstimulated WT models as reference), and enzyme-linked immunoassay. (**A**) A compact epidermis forms in WT cells with and without IFN-α stimulation, whereas a loose arrangement is apparent when *ISG15^–/–^* cells are used. Visualization of the epidermis is enhanced by IHC staining for TGF-β1 (brown). Scale bars: 50 μm. (**B**) Lower TGF-β1 staining intensity in the epidermal layer of the *ISG15^–/–^* 3D model (based on automated image analysis of TGF-β1 IHC). A representative example comparing staining with specific and nonspecific (isotype control) antibody is shown in Supplemental Figure 7. (**C** and **D**) Desmosomes (identified by red arrows) are easily identified by transmission electron microscopy in the WT, but not in the *ISG15^–/–^* 3D model. Scale bars: 2 μm. (**D**) Markedly lower desmosome density in the *ISG15^–/–^* 3D model after IFN-α stimulation, as determined by 2 independent examiners by manual counting of typical structures in electron microscopic images.

**Figure 8:**
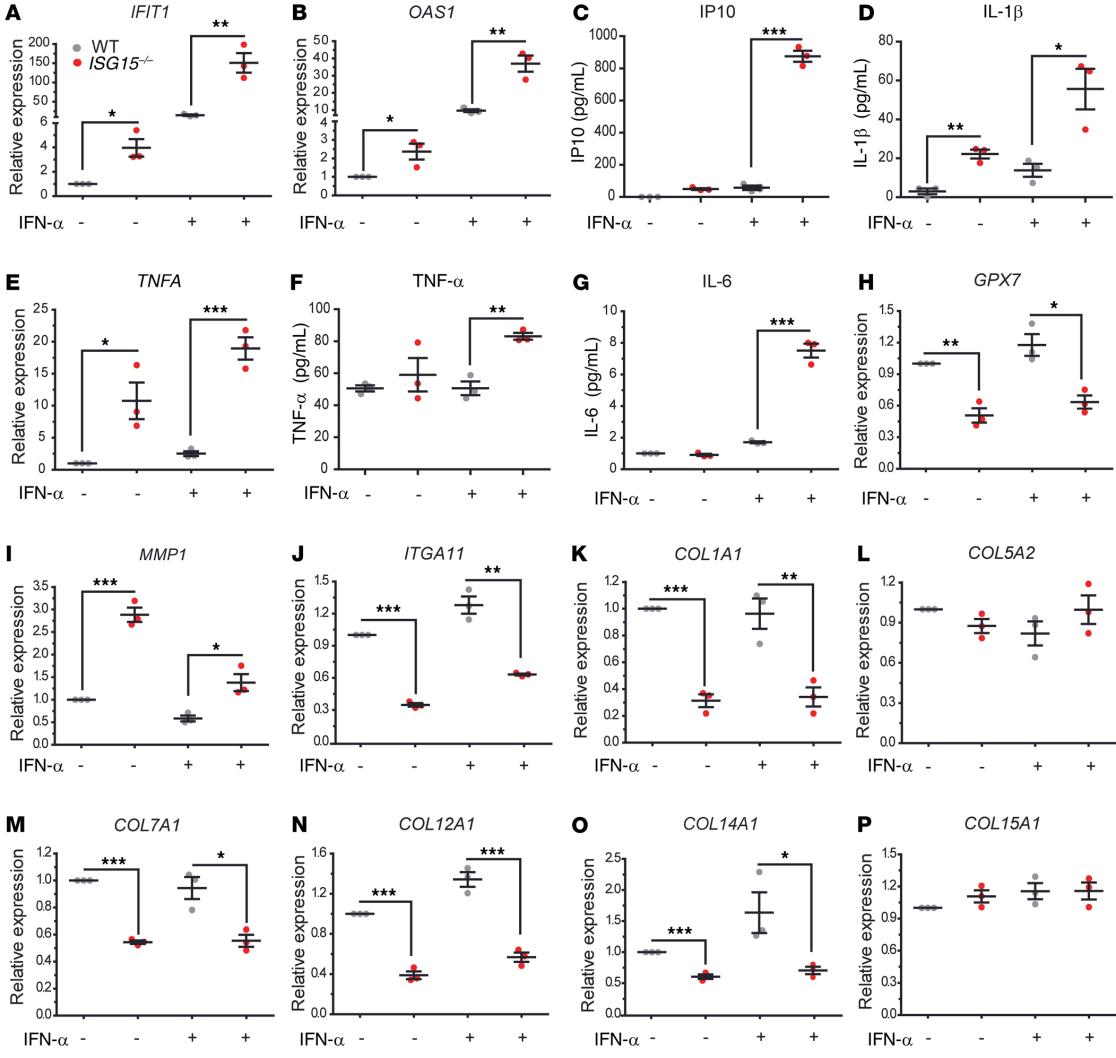
Hyperinflammation and dysregulated epidermal homeostasis in the *ISG15^–/–^* 3D epidermis model. The 3D epidermis model was performed as in Figure 7. Expression of target mRNAs in tissue was measured by RT-qPCR, and cytokine concentrations in supernatant by enzyme-linked immunoassay. (**A**–**G**) Increased expression of *IFIT1*, *OAS1*, and *TNFA* mRNA and IL-1β protein was seen in the unstimulated model, whereas increased IP10, TNF-α, and IL-6 levels were seen only after IFN-α stimulation. (**H**–**J**) Decreased expression of *GPX7* (an antioxidant factor) and integrin *ITGA11* mRNA but increased expression of *MMP1* mRNA in the *ISG15^–/–^* 3D model. (**K**–**P**) Decreased expression of several collagen mRNA species but not *COL5A2* and *COL15A1* in the *ISG15^–/–^* 3D model. *n* = 3, ± SEM; *P < 0.05, **P < 0.01, ***P < 0.001 (Student’s *t* test).

There are fundamental differences in ISG15 function between mice and humans, notably in that USP18 stabilization does not depend on ISG15 in mice (22). Moreover, *ISG15^–/–^* mice do not develop skin lesions. We therefore used a human ex vivo epidermis model to characterize the consequences of loss of ISG15 at the tissue level. HaCaT is an immortalized keratinocyte cell line that has the ability to differentiate and form a stratified epidermal structure (31). When used in a 3D model of epidermis formation, HaCaT cells formed an epidermis-like structure (Figure 7A). Notably, *ISG15^–/–^* 3D models showed a less compact and less organized arrangement of cells in the epidermis, indicating a defect in cell-cell adhesion. IHC also revealed a reduction of epidermal TGF-β1 staining in the epithelial layer of the *ISG15^–/–^* 3D models (Figure 7B), which agreed well with its reduction in the cell-based analyses shown in Figure 4. Ultrastructural examination of the 3D models by transmission electron microscopy revealed a prominent reduction in desmosome number per unit area (Figure 7, C and D), which was consistent with the decreased expression of cell adhesion molecules in *ISG15^–/–^* HaCaT cells detected by microarrays. The *ISG15^–/–^* 3D models also exhibited increased levels of mRNA encoding ISGs and proinflammatory cytokines (*CXCL10*, *TNFA*, and *IL6*) along with a reduction in the ROS scavenger *GPX7* (Figure 8, A–H). In addition, there was upregulation of *MMP1* and downregulation of collagens and integrin (*ITGA11*) in the *ISG15^–/–^* 3D epidermis models (Figure 8, I–P). Together, these findings suggested that ISG15 deficiency in the 3D models led to hyperinflammation, collagen dysregulation, and defects in cell adhesion molecules and desmosome formation, and that this effect was exacerbated by prolonged exposure to IFN-α.

### A conjugation-deficient ISG15 variant rescues the inflammatory phenotype but not collagen imbalance in ISG15^–/–^ fibroblasts.

**Figure 9:**
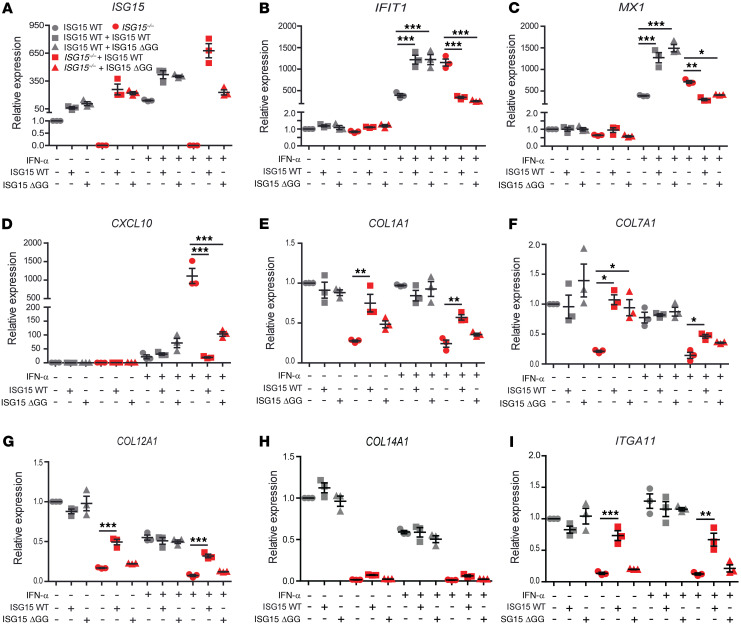
WT ISG15 rescues the WT phenotype in *ISG15^–/–^* fibroblasts, but conjugation-deficient ISG15 only normalizes IFN-I responses. *ISG15^–/–^* or WT fibroblasts were stably transduced with lentiviral vectors expressing either WT ISG15 or an ISG15 variant deficient in conjugation as a result of a 2–amino acid deletion (ΔGG). Cells were stimulated with IFN-α (1000 IU/mL) for 24 hours as indicated. (**A**–**I**) Expression analysis of the indicated target mRNA by RT-qPCR (ΔΔCt method, using unstimulated WT cells used as reference). WT ISG15 rescued expression of all targets except *COL14A1*, whereas the ΔGG mutant rescued only expression of *IFIT1*, *MX1*, and *CXCL10*. Statistical significance of rescue by WT versus ΔGG is also summarized in Table 1. *n* = 3, ± SEM; *P < 0.05, **P < 0.01, ***P < 0.001 (1-way ANOVA with Tukey’s post hoc test).

In order to test to what extent the phenotype of the *ISG15^–/–^* fibroblasts was due to lack of the ISGylation activity of ISG15, we used *ISG15^–/–^* fibroblasts stably expressing WT ISG15 or the conjugation-deficient mutant ISG15 ΔGG. Transduction with the WT construct by and large normalized expression of ISGs, collagens (*COL1A1*, *COL7A1*, *COL12A1*), and *ITGA11*, but — curiously — had no effect on *COL14A1* expression. ISG15 ΔGG efficiently rescued aberrant ISG expression (*MX1*, *IFIT1*, *CXCL10*), but had no significant effect on collagen and *ITGA11* expression (Figure 9 and Table 1). These data suggest that ISG15-dependent negative regulation of ISG expression (via stabilization of USP18) is relatively independent of its ISGylation activity, whereas ISGylation is required for physiological regulation of collagen and integrin expression. The latter could potentially be explained by a model in which the absence of ISGylation leads to loss of posttranslational stabilization of TGF-β1, which in turn leads to collagen deficiency in *ISG15^–/–^* fibroblasts.

### Effects of bioactive molecules on inflammation and collagen dysregulation in ISG15-deficient dermal fibroblasts.

In order to identify a potential treatment for skin ulcerations due to ISG15 deficiency, we evaluated dexamethasone (DEXA), ruxolitinib (RUX; a JAK1/2 inhibitor that diminishes IFN-I signaling; ref. 32), doxycycline hyclate (DOXY), and TGF-β1. We tested DEXA because treatment with corticosteroids had some efficacy in the 2 index cases. As expected, DEXA (0.05–50 μM) reduced *CXCL10* levels in WT and *ISG15^–/–^* fibroblasts, but it failed to normalize *IFIT1* and *COL1A1* mRNA levels (Supplemental Figure 8, A–C). Treatment of WT and *ISG15^–/–^* fibroblasts with RUX at concentrations of 0.5–5 μM resulted in a significant reduction in *IFIT1* and *CXCL10* mRNA, but it failed to reverse *COL14A1*, *COL1A1*, and *MMP1* levels. In fact, *MMP1* levels actually increased at the lowest RUX dose and only decreased at higher doses (Supplemental Figure 8, D–H). In addition to its antibiotic effects, DOXY can inhibit MMP1 expression and activity (33, 34); as expected, 50 μM DOXY produced a mild reduction in *MMP1* as well as an induction of *COL1A1* mRNA levels (Supplemental Figure 9, A and B). Moreover, since we had observed downregulation of TGF-β1 in *ISG15^–/–^* cells, we evaluated the efficacy of TGF-β1 in restoring collagen levels. Addition of TGF-β1 did not lead to pronounced changes in *IFIT1* mRNA expression, but caused significant downregulation of *MMP1* and upregulation of *TIMP1* and *COL1A1* mRNAs (Supplemental Figure 9, C–F). Considering the variable effects of the single treatments, we then evaluated combination treatments for potential synergy. RUX + DOXY and RUX + TGF-β1 equally reduced hyperinflammation, whereas RUX + TGF-β1 resulted in greater reduction in *MMP1* and increase in *COL1A1* and *COL7A1* (Supplemental Figure 9, G–K). Notably, these treatments did not lead to a complete normalization of the parameters assayed, but led to increased collagen transcription also in the WT cells. Moreover, the triple treatment did not confer an additional benefit in this cellular model, and it failed to reduce the increased cellular and mitochondrial ROS levels in the *ISG15^–/–^* fibroblasts (Supplemental Figure 10).

### Effects of combination treatment with RUX, DOXY, and TGF-β1 on ISG15^–/–^ HaCaT keratinocytes.

Enrichment analysis showed that triple treatment of IFN-α–stimulated *ISG15^–/–^* HaCaT cells depleted GO terms relating to IFN-I responses and other antiviral responses and that, notably, collagen metabolic process and extracellular matrix organization were the most significantly enriched GO terms (Supplemental Figure 11A). Among other mRNAs, triple treatment led to upregulation of the desmosome constituents *DSC2* and *DSG3*, *FGF2* (a master regulator of wound healing), and *CXCL8* (a chemokine that attracts neutrophils to initiate wound healing; ref. 35), and *TNFAIP3* (also known as *A20*), which inhibits NF-κB activity and has broad antiinflammatory properties (36) (Supplemental Figure 11B).

### Collagen turnover is increased in ISG15^–/–^ fibroblasts and HaCaT keratinocytes and decreases under treatment with RUX/DOXY/TGF-β1.

**Figure 10:**
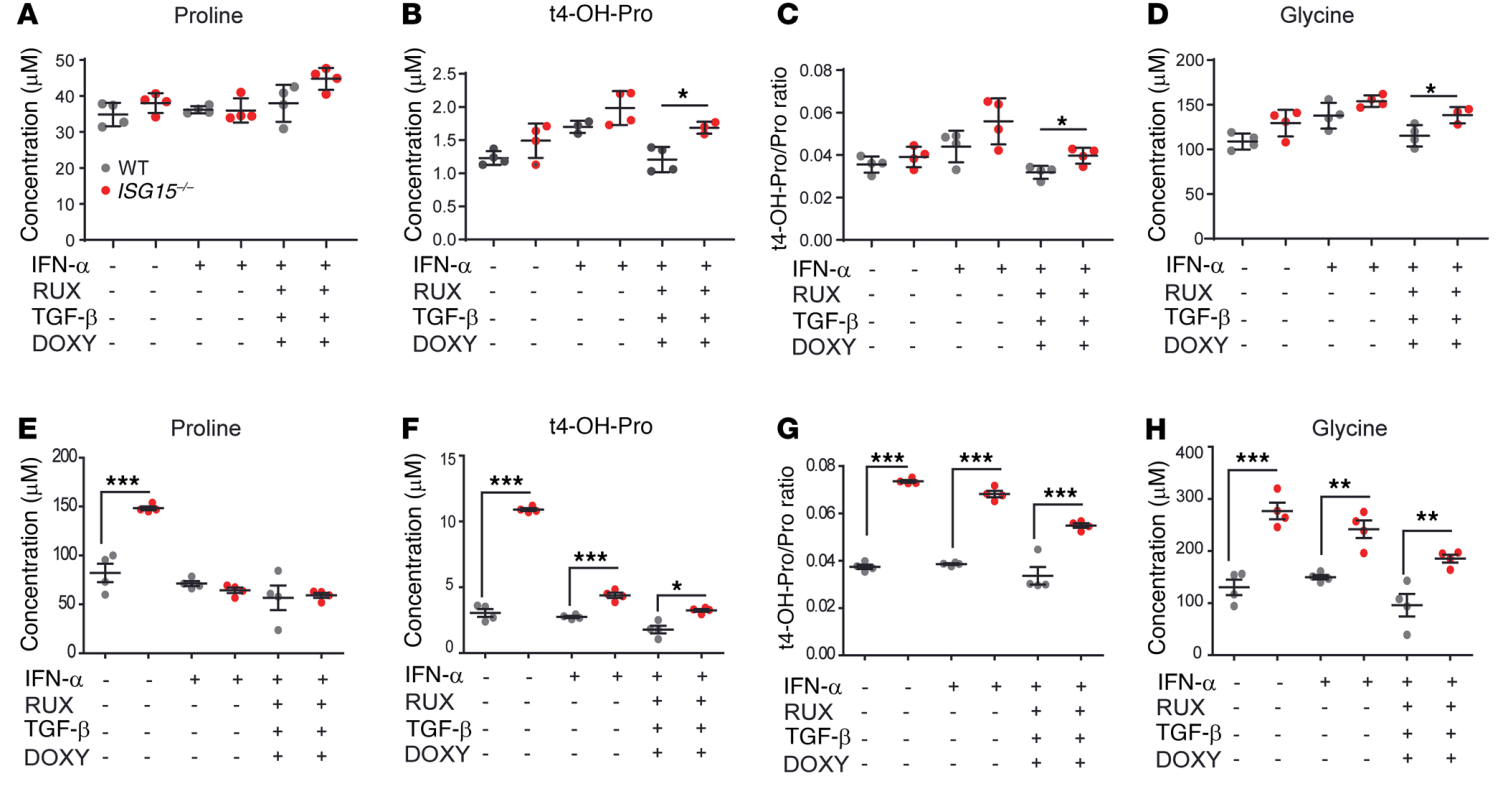
Increased collagen turnover in immortalized fibroblasts and *ISG15^–/–^* HaCaT keratinocytes. WT and *ISG15^–/–^* immortalized fibroblasts or HaCaT keratinocytes were stimulated for 8 hours with IFN-α (1000 IU/mL) as indicated and the stimulated cells also incubated for 16 hours with triple treatment consisting of ruxolitinib (RUX; 0.5 μM), TGF-β (10 nM), and doxycycline (DOXY; 50 μM). Concentrations of proline, 4-OH-proline, and glycine in cell pellets were measured by liquid chromatography–mass spectrometry. (**A**–**D**) Fibroblasts. There are tendencies of increased t4-OH-proline and glycine levels and t4-OH/proline ratio in the IFN-α–stimulated *ISG15^–/–^* cells, which are reduced by triple treatment to levels similar to those in untreated IFN-α–stimulated WT cells. (**E**–**H**) HaCaT keratinocytes. Elevated proline concentrations were detected in unstimulated *ISG15^–/–^* cells only (**E**). 4-OH-proline concentrations were consistently higher in *ISG15^–/–^* cells but decreased with treatment to levels found in stimulated WT cells (**F**), whereas the 4-OH-proline/proline ratio (a marker for collagen turnover) (**G**) and glycine concentrations (**H**) approached WT levels only partially. *n* = 3, ± SEM; *P < 0.05, **P < 0.01, ***P < 0.001 (Student’s *t* test with Bonferroni correction; WT vs. *ISG15^–/–^*).

Increased free levels of the major amino acid constituents of collagen (4-OH-Pro, Pro, Gly) are indicators of increased collagen turnover. This is particularly true for 4-OH-Pro, which is only formed after initial incorporation of Pro into a collagen chain, and the 4-OH-Pro/Pro ratio is considered a biomarker for collagen turnover (37). We therefore compared their concentrations in WT and *ISG15^–/–^* fibroblasts and HaCaT keratinocytes and assessed changes under IFN-α stimulation and triple treatment. In fibroblasts, there was a trend toward higher t4-OH-Pro concentrations, t4-OH/Pro ratio, and Gly concentrations in the *ISG15^–/–^* cells, and treatment reduced t4-OH-Pro, Gly, and t4-OH/Pro ratio values to those seen in untreated IFN-α–stimulated WT cells (Figure 10, A–D). In HaCaT cells, concentrations of all 3 were higher in the *ISG15^–/–^* cells, and all returned close to concentrations found in the untreated IFN-α–stimulated WT cells upon triple treatment (Figure 10, E–H). Nonetheless, the t4-OH/Pro ratio remained partially elevated in *ISG15^–/–^* cells, indicating that collagen homeostasis improved but did not normalize completely under treatment.

### Combined treatment with RUX/DOXY/TGF-β1 reverses inflammation and collagen and DSG4 deficiency and improves epidermis formation in the ISG15^–/–^ 3D skin model.

**Figure 11:**
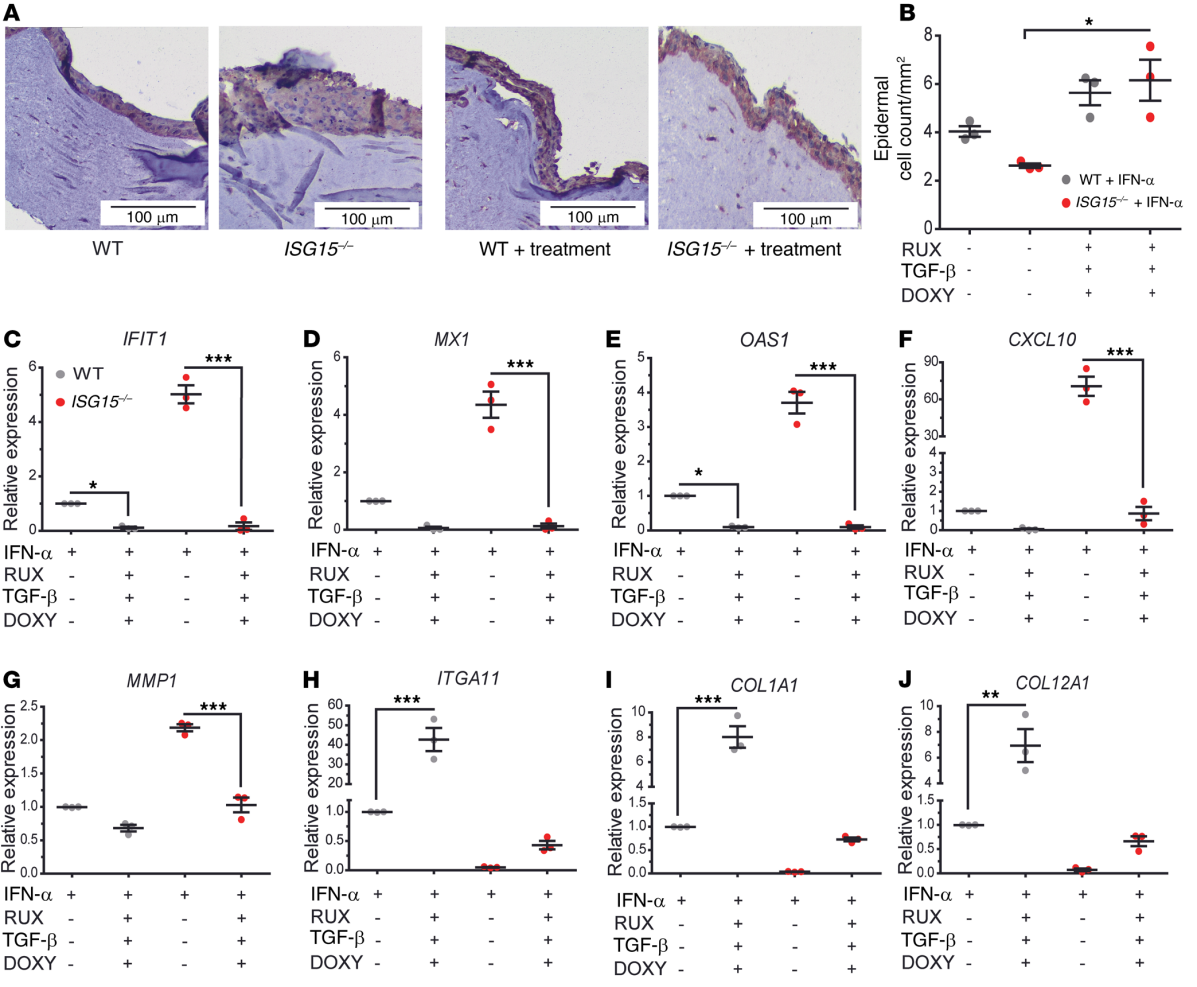
Combined treatment with RUX, DOXY, and TGF-β1 normalizes epidermis formation, inflammation, and collagen synthesis in the *ISG15^–/–^* 3D model. 3D epidermis models were generated as in Figure 7 and cultured in the presence of RUX (0.5 μM), DOXY (50 μM), and TGF-β (10 nM) from day 8 to day 14. (**A**) H&E stains showing thicker, less compact epithelial layer in the *ISG15^–/–^* model, which appears to normalize under treatment. Scale bars: 100 μm. (**B**) Triple treatment leads to increase of epithelial cell density of the *ISG15^–/–^* model to WT density. (**C**–**J**) Triple treatment reduces expression of IFN-regulated mRNAs (**C**–**F**) and *MMP1* (**G**), and increases expression of *ITGA11* (**H**), *COL1A1* (**I**), and *COL12A1* (**J**). *n* = 3, ± SEM; *P < 0.05, **P < 0.01, ***P < 0.001 (Student’s *t* test).

In the 3D model, the triple treatment effectively reduced the increased epithelial layer thickness of the *ISG15^–/–^* model to that of the WT model (Figure 11A) and also apparently normalized cell-cell adhesion, as cell densities were similar between the treated WT and *ISG15^–/–^* 3D models (Figure 11B). Compared with the variable effects on expression of collagens and inflammatory mediators seen in the cellular models (Supplemental Figures 8 and 9), the triple treatment was markedly more effective in the *ISG15^–/–^* 3D model: all parameters assayed approached or reached expression seen in the untreated WT model, and the overshooting responses in collagen transcription in the *ISG15^–/–^* fibroblasts (Supplemental Figure 9) were not seen (Figure 11, C–J). Collagen expression could not be verified at the protein level because of the collagen matrix present in this model. We therefore assessed levels of DSG4, a constituent of desmosomes. As expected, DSG4 levels were lower in the *ISG15^–/–^* model both with and without IFN-α stimulation. Both single RUX treatment and the combination treatment raised DSG4 levels close to the expression seen in the IFN-α–stimulated untreated WT 3D model (Supplemental Figure 12A). However, WT desmosome densities were not restored (Supplemental Figure 12B), suggesting that the treatment did not activate all steps for the formation of morphologically verifiable desmosomes.

### Treatment with RUX but not TGF-β1 corrects impaired cell migration in ISG15^–/–^ cells.

**Figure 12:**
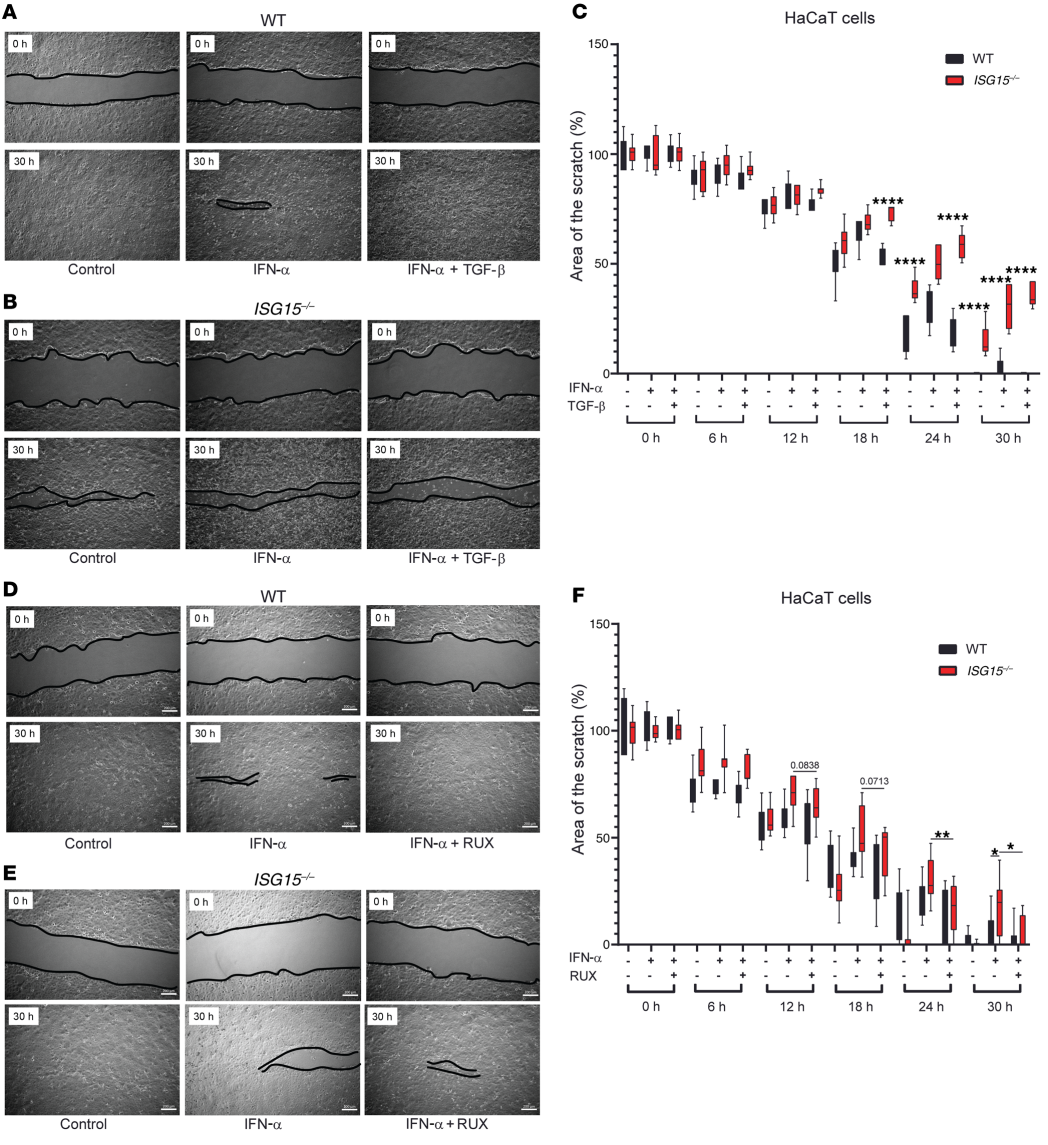
Retarded cell migration in *ISG15^–/–^* HaCaT keratinocytes normalizes under treatment with RUX but not TGF-β1. Scratch assays were performed with WT or *ISG15^–/–^* HaCaT keratinocytes, and gap closure was measured by automated image analysis for 30 hours. Images show start (0 hours) and end (30 hours). Images of all time points are shown in Supplemental Figures 15 and 16. The black lines delineate the borders of the scratch. (**A**–**C**) TGF-β1 treatment. Treatment delays gap closure similarly to triple treatment (see Supplemental Figure 14). (**D**–**F**) RUX treatment. Treatment accelerates gap closure in *ISG15^–/–^* cells to a speed similar to that in WT cells. *n* = 3, ± SEM; *P < 0.05, **P < 0.01, ***P < 0.001 (1-way ANOVA with Tukey’s post hoc test).

The index cases did not manifest impaired healing of noninflamed wounds, but the enrichment analysis had shown enrichment of pathways relating to cell migration and regulation of response to wound healing in the *ISG15^–/–^* HaCaT cells. We therefore assessed whether a defect in cell migration might contribute to the *ISG15^–/–^* phenotype. Scratch tests were performed on *ISG15^–/–^* and WT fibroblasts and HaCaT cells with and without the combination treatment. *ISG15^–/–^* fibroblasts demonstrated impaired migration both with and without IFN-α stimulation, but the triple treatment impaired migration further (Supplemental Figure 13). In HaCaT cells, the defect in migration was more apparent in IFN-α–stimulated cells, and the triple treatment impaired migration more strongly in the *ISG15^–/–^* cells (Supplemental Figure 14). Considering the well-documented cytostatic effects of TGF-β1, single TGF-β1 treatment was applied to scratch tests performed with HaCaT cells. Indeed, TGF-β1 delayed gap closure in the IFN-α–stimulated *ISG15^–/–^* cells (Figure 12, A–C, and Supplemental Figure 15). In contrast, single treatment with RUX improved gap closure by *ISG15^–/–^* cells at the later time points to values comparable to those for untreated WT cells (Figure 12, D–F, and Supplemental Figure 16). Thus, impaired cell migration is part of the cellular phenotype of ISG15 deficiency and largely responds to control of elevated IFN-I signaling. Considering the results of the 3D model and the cell migration studies together, it appears that a combined therapy with RUX/DOXY/TGF-β1 is a potential treatment for skin ulcerations in ISG15 deficiency, but that the effects of TGF-β1 on cell migration require clarification.

## Discussion

Skin lesions have only recently been appreciated as part of the clinical spectrum of ISG15 deficiency (13, 14). We describe two children with complete loss of function of ISG15 and skin ulcerations as the predominant phenotype, and our results suggest that the underlying pathology is due to ISG15’s well-known role of limiting inflammation plus previously unrecognized functions in maintaining cell migration and connective tissue and epidermis homeostasis.

### Potential mechanisms.

Even though there were some differences between fibroblasts, ECs, and keratinocytes, we found a recurring pattern of hyperinflammation, decreased synthesis of collagens and cell adhesion molecules, and dysregulated synthesis of MMPs in the 3 cell types as well as the 3D epidermis model. The critical contribution of dysregulated collagen synthesis and dysfunction of fibroblasts and keratinocytes to the phenotype is underscored by the cosmetically disfiguring scars that formed in the patients when lesions healed under immunosuppressive therapy. The increased ROS levels, paralleled by decreased expression of ROS scavengers like GPX7, likely further contribute to the propensity for skin ulcerations, for instance by inducing serine proteases and MMPs, which can negatively affect ECM and wound repair (38). Similarly, oxidative stress can induce MMP1 expression by activating the MEK/ERK pathway (39). However, ROS levels were not elevated in *ISG15^–/–^* HaCaT cells even after IFN-α stimulation, and triple treatment with RUX/DOXY/TGF-β1 did not reduce the elevated ROS levels in *ISG15^–/–^* fibroblasts. Moreover, we actually observed reduced levels of MMP1 in *ISG15^–/–^* HaCaT cells. In a separate study we recently found that loss of ISG15 leads to elevated ROS levels in IFN-α–stimulated *ISG15^–/–^* macrophages, but that ROS levels normalize under RUX treatment (Waqas et al., unpublished observations). Taken together these results suggest that (a) ROS responses in ISG15 deficiency differ depending on the cell type and (b) may not be causally involved in all aspects of the cellular phenotype, but rather form part of a complex regulatory network.

In the present work we observed that knocking down TGF-β1 expression or inhibiting PI3K activity reproduced some aspects of ISG15 deficiency. In this model, reduced phosphorylation of AKT could be one pathway to the observed lower TIMP1 expression (40). It is important to note, though, that both interventions led to additional changes in *ISG15^–/–^* cells, demonstrating that the TGF-β1 and p-AKT–mediated effects are only partially ISG15 dependent. PI3K inhibitors are finding increasing use in clinical medicine. Considering these additional effects in the *ISG15^–/–^* cells, PI3K inhibitors may actually exacerbate certain clinical aspects of ISG15 deficiency such as skin lesions.

Notably, of the *ISG15^–/–^* phenotype, the conjugation-defective ISG15 ΔGG variant largely rescued hyperinflammation but did not rescue decreased expression of collagens and the adhesion molecule ITGA11. This is consistent with well-documented experience that hyperinflammation in ISG15 deficiency is due to loss of USP18 (which inhibits IFNAR signaling): stabilization of USP18 by ISG15 is mediated by free ISG15 and does not require the conjugation function of ISG15 (8). On the other hand, the results indicate that the underlying physiological role(s) of ISG15 in collagen and integrin expression at least in part require ISGylation of upstream factors in the respective regulatory pathways. ISG15 has been shown to form complexes with up to several hundred cellular proteins (41), and it will now be important to identify those ISG15 targets that are critical for collagen synthesis and epidermis integrity. Moreover, considering that ulcerating skin lesions are a frequent feature of type I interferonopathies that are not due to ISG15 deficiency and thus support intact ISGylation, it remains to be solved to what extent hyperinflammation versus dysregulation of epidermal homeostasis contributes to the frequency, type, and severity of these lesions.

We identified retarded cell migration as an additional cellular phenotype of ISG15 deficiency. Functionally, this agrees with observations from cancer research that overexpression of ISG15 in tumor cells is associated with increased cell migration (42). Clinically, our two patients did not manifest overt signs of impaired healing of wounds in the absence of inflammation. For instance, the circumcision of the boy healed without complications. However, impaired cell migration may contribute to the observed severity of their inflammation-associated lesions and the associated conspicuous scar formation.

### Implications for treatment.

Both patients experienced recurring flares of skin lesions that would remit only after aggressive immunosuppressive treatment including cyclophosphamide in P1, and especially P2 developed the well-known untoward effects of chronic corticosteroid treatment. Treatment with JAK1/2 inhibitors such as RUX is emerging as a treatment for patients with type I interferonopathies (43, 44). Our cellular studies demonstrated that hyperinflammation and reduced cell migration of ISG15 deficiency responded well to RUX; however, the connective tissue–related defect did so only partially, and adding doxycycline and TGF-β1 had incomplete additional benefits in cells. The efficacy of this triple treatment in the 3D epidermis model, however, was striking and was not entirely expected, considering the somewhat heterogeneous effects in the cellular experiments. Most likely, it is the ability of the 2 cell types to interact in a functional network that provided the basis for the greater responsiveness in the 3D model. Nonetheless, even though DSG4 levels increased under single RUX and triple treatment, WT densities of desmosomes could not be restored, indicating the need for further optimization.

### Does ISG15 deficiency feature cutaneous vasculitis?

Skin lesions in interferon-driven disorders are often vasculitic in nature (3, 15). Histological findings in our 2 cases were relatively nonspecific and did not reveal evidence of vasculitis, but rather perivascular inflammation. On the other hand, the macroscopic appearance of the lesions of both index cases is consistent with a cutaneous vasculitis. Histological vasculitis was also not documented in the other recent report of skin lesions in ISG15 deficiency in which skin tissue was analyzed (13); it is therefore possible that the histological extent of vasculitis is not very severe and affected areas may be missed when a single punch biopsy is taken. Considering the observed dysfunction of multiple cell types that normally maintain skin integrity (keratinocytes, fibroblasts, vascular ECs), coupled with a defect in cell migration, it is tempting to speculate that due to compromised epidermal integrity and reduced survival of underlying tissue, tissue necrosis ensues without the marked hypoperfusion and hypoxia that usually result from a severe small-vessel vasculitis. Analysis of tissue samples from additional patients is necessary to clarify this issue.

### Limitations of the study.

We employed HaCaT cells as a commonly used keratinocyte component of the epidermis model. It has been shown that N/TERT-immortalized keratinocytes correspond closely to primary human keratinocytes in this model (45). Their use might have given additional insights from this model, in particular if we could have applied the technique to keratinocytes from patient skin samples. For sociocultural reasons, it was not possible to obtain additional blood or tissue samples from the patients for investigational purposes. We could therefore not improve the 3D model in this respect. We also could not assess interferon expression in patient blood to confirm a laboratory diagnosis of type I interferonopathy by the characteristic IFN signature (46). This would have been helpful since, despite the complete absence of ISG15 expression, neither patient manifested other common features of ISG15 deficiency such as severe mycobacterial infection or seizures, even though CNS calcifications were found in P1 (brain CT was not performed on P2).

Even though skin lesions appear to occur in nearly all individuals with ISG15 deficiency, those in our patients were the most extensive ones and were also unusual in the strong degree of scar formation. Surprisingly, the single-nucleotide variant analysis revealed nonsynonymous polymorphisms in several genes with documented roles in skin or connective tissue in both index cases, and the gene deletion analysis identified a deletion in a gene involved in oxidative stress responses in P1. For reasons of data protection we could not compare these findings to genome sequences of other ISG15 patients with and without cutaneous manifestations. Nonetheless, one may speculate that the severe phenotype of our patients is in part due to some of these potential modifier genes. Clearly, much further work is required to understand the genomic features driving the spectrum of clinical manifestations of ISG15 deficiency. Taken together, our results suggest that the contribution of ISG15 deficiency to skin homeostasis varies from cell type to cell type and that, despite the monogenic nature of this disorder, its final phenotype is the result of a complex network of functional interactions.

## Methods

### Genomic and genetic analyses.

See Supplemental Methods.

### Histology.

H&E staining was performed using standard procedures. ISG15 protein was stained by 3-step IHC using ISG15 primary antibodies directed against the C-terminus (aa 136–165; LS-C98671, LSBio; polyclonal rabbit IgG) and the ZytoChem kit (Zytomed Systems) for secondary antibody and color development. Preimmune rabbit IgG at the same concentration was used as negative control. For TGF-β1 IHC, mouse monoclonal anti–human TGF-β1 IgG1 (clone 7F6, Novus Biologicals) was used, and nonspecific mouse IgG1 (X0931, Dako/Agilent) was used at the same concentration as negative control. The Envision Kit (Dako/Agilent) was applied for secondary antibody and color development. IHC staining intensity was quantified using an Olympus BH-2 microscope equipped with an Olympus sc-100 camera (Olympus) and cellSense Dimension software version 1.8.1 (Olympus).

### Transmission electron microscopy.

See Supplemental Methods.

### Cell culture and reagents.

hTERT-immortalized dermal fibroblasts were grown in DMEM supplemented with 100 μg/mL penicillin-streptomycin (10,000 U/mL), 1% Glutamax (all GIBCO Life Technologies), and 10% FCS (Sigma-Aldrich). hiPSCs and hiPSC-derived vascular endothelial cells (hiPSC-ECs) were cultured as described previously (47). Briefly, hiPSCs were maintained under feeder-free conditions on Geltrex-coated tissue culture flasks (TPP) in mTeSR1 medium (Stem Cell Technologies) and passaged as single cells using Accutase (PAA Laboratories) with a seeding density of 3.6 × 10^4^ cells/cm^2^. To induce EC differentiation, hiPSC cultures were treated with 25 ng/mL BMP4 (Bio-Techne) and the WNT pathway activator CHIR 90221 (7.5 μM) in N2B27 medium (Thermo Fischer Scientific) for 2 days without medium change. From day 3 to day 7, cultures were maintained in StemPro-34 medium (Thermo Fischer Scientific) supplemented with 260 ng/mL rhVEGF-A165 and 2 μM forskolin (Sigma-Aldrich) with daily medium change. On day 7 of differentiation, CD31-positive cells were purified by Magnetic Activated Cell Sorting using CD31 MicroBead Kit (Miltenyi Biotec). hiPSC-ECs were then cultured in ECGM-2 medium (PromoCell) on plates coated with fibronectin (Corning). The human keratinocyte cell line HaCaT was cultured in RPMI 1640 medium (GIBCO Life Technologies) supplemented with 30% FCS, 100 μg/mL penicillin-streptomycin, 500 μg/mL gentamycin, and 1% each Glutamax and nonessential amino acid solution (NEAA; GIBCO Life Technologies). Human primary keratinocytes were grown in Keratinocyte Growth Medium 2 with Supplement Mix (bovine pituitary extract, epidermal growth factor, insulin, and transferrin) and 0.06 mM CaCl_2_ (all PromoCell).

### Generation of ISG15-knockout and -knockin cells.

hTERT-immortalized dermal fibroblasts from ISG15-deficient patients and WT controls, as well as patient and control fibroblasts stably transduced with a vector expressing ISG15, conjugation-deficient ISG15 ΔGG, or luciferase, have been described previously (22). The stable expression of the transduced constructs was periodically checked by Western blotting. In HaCaT cells and hiPSCs, the *ISG15* locus was ablated with the CRISPR/Cas9 system using 2 guide RNAs (gRNAs) cloned separately into vector PX458_pSpCas9-2A-GFP (Addgene): gRNA1, GCGCAGATCACCCAGAAGAT; and gRNA2, GGTAAGGCAGATGTCACAGG. For HaCaT cells, 1 × 10^6^ cells were transfected with 1 μg of each targeting plasmid using Lipofectamine 3000 (Thermo Fischer Scientific), and green fluorescent protein–positive (GFP-positive) cells were isolated the next day by fluorescence-activated cell sorting. The cell suspensions were then serially diluted (1:10) in order to obtain single-cell clones. We used the MHHi001-A cell line (48) for the *ISG15* knockout in hiPSCs. Briefly, 2.75 μg of each plasmid was used to transfect 1 × 10^6^ iPSCs with Lipofectamine 3000. Three days after transfection the cells were sorted for GFP expression and seeded in low density on irradiated mouse embryonic fibroblasts, and arising colonies were picked manually and expanded clonally. Homozygosity of the *ISG15* knockout was confirmed by PCR analysis of the genomic DNA, resulting in a single band of 378 bp in homozygous knockout clones (fwd_TTTCTTCCGCTCACTCTGGG, rev_GTTCGTCGCATTTGTCCACC) and the absence of the 5′ (fwd_TTTCTTCCGCTCACTCTGGG, rev_GAGGATCTCAGGGGTGACCT) and 3′ (fwd_AGAGGACAGACAGGAGGGAG, rev_GTTCGTCGCATTTGTCCACC) junction of the WT allele. Lack of ISG15 protein expression in the *ISG15^–/–^* HaCaT cells and iPSC-derived ECs was verified by Western blot (13).

### Mass spectrometry–based proteomics.

See Supplemental Methods.

### Amino acid measurements by mass spectrometry.

Cell extracts were prepared as described in ref. 49. See Supplemental Methods for details.

### Cell transfection.

Human *ISG15^–/–^* dermal fibroblasts were seeded at a density of 0.4 × 10^6^ cells per well in 6-well plates, and the next day 2 μg each of empty vector (pcDNA 6.0) or vector containing *ISG15*-WT or *ISG15*-mutant (c.288C>G) cells was transfected using Lipofectamine LTX reagent (Thermo Fisher Scientific) according to the manufacturer’s protocol. Cells were harvested in RIPA buffer (50 mM Tris [pH 8.0; Thermo Fisher Scientific], 150 mM NaCl [Th. Geyer GmbH & Co.], 0.1% SDS [Merck], 0.5% sodium deoxycholate [Carl Roth], and 1% Nonidet P-40 [Sigma-Aldrich]) containing complete Mini Protease Inhibitor Cocktail (Roche) and PhosSTOP tablets (Sigma-Aldrich) for immunoblots.

### siRNA-mediated silencing.

In order to knock down *ISG15* mRNA, 25 nM siRNA targeting *ISG15* or scrambled control siRNA (Sigma-Aldrich) or 25–100 nM siRNA targeting *TGFB1* (ON-TARGETplus Human TGFB1 siRNA, SMARTpool, Horizon Discovery) or scrambled control was transfected into cells for 48 hours at 37°C using Lipofectamine RNAiMAX reagent (Invitrogen) according to the manufacturer’s protocol.

### Cell migration assays (scratch test).

Cells were seeded in 6-well plates at a density of 3 × 10^5^ cells per well. Once all the wells were 90%–100% confluent, a straight scratch was made with a 200 μL pipette tip held at a 45° angle with a single steady motion across the well. Cells were washed with 1× PBS to remove dead or floating cells. Afterward, cells were stimulated with IFN-α with or without ruxolitinib, doxycycline, and/or TGF-β treatment prepared in fresh HEPES-buffered culture media. Interwell spaces of the cell culture plate were filled with sterile distilled water to minimize evaporation for the duration of the assay. The plate was placed in an Axio Observer Z1 inverted microscope (Carl Zeiss) with incubation chamber at 37°C, 5% CO_2_ in a humidified environment. Images were captured with the ×5 objective at specific time intervals and analyzed and exported using Zen 3.0 software (Zeiss).

### 3D epidermis model.

In vitro epidermis equivalents were prepared as described before (50) with minor modifications. Briefly, 5 × 10^5^ fibroblasts in 1 volume of FCS per gel were mixed with 8 volumes of collagen (3 mg/mL; Pure Col, Advanced BioMatrix) and 1 volume of 10× HBSS (GIBCO/Invitrogen). For each epidermis equivalent, 2.5 mL of the gel was poured into 6-well cell culture inserts (BD Falcon) in a deep 6-well tray. After gelation in a humidified atmosphere at 37°C (without CO_2_) for 2 hours, the gels were equilibrated with Keratinocyte Growth Medium (KGM; Lonza) for another 2 hours at 37°C/5% CO_2_. The medium on the top of the insert was then removed, and 1 × 10^6^ HaCaT cells in 2 mL of KGM per gel were added to the inserts. After incubation overnight, the medium was removed from the inserts and the external well. The medium in the external well was replaced by a modified KGM without bovine pituitary extract, but supplemented with 1.3 mM CaCl_2_ (PromoCell), 10 μg/mL transferrin, 50 μg/mL ascorbic acid (both Sigma-Aldrich), and 1 mg/mL BSA for the air-liquid interphase culture. No medium was added on the surface of the epidermis equivalents during the following cultivation phase. The medium was changed every other day for up to 14 days.

### Cellular and mitochondrial ROS measurements.

Cells were seeded at a density of 2 × 10^5^ cells per well in 12-well plates, incubated overnight, and then stimulated with IFN-α for 24 hours. For detection of cellular ROS, medium was removed and fresh medium containing 25 μM of the cell membrane–permeable fluorescent probe 6-carboxy-2′,7′-dichlorodihydrofluorescein diacetate (carboxy-H2DCFDA; C-400, Molecular Probes, Invitrogen) was added and incubated for 30 minutes at 37°C. For mitochondrial ROS, medium containing 5 μM MitoSox Red (mitochondrial superoxide indicator; Invitrogen) was added and incubated for 10 minutes at 37°C. After the appropriate incubation times, ROS dyes were removed, and cells were washed with PBS and then harvested in ice-cold PBS for flow cytometry. Cellular and mitochondrial ROS were measured via FL-1 and FL-2 channels, respectively, using a FACSCalibur flow cytometer (BD Biosciences).

### RT-qPCR analysis.

See Supplemental Methods.

### Western blotting.

See Supplemental Methods.

### Determination of autoantibodies.

Screens for anti-nuclear antibodies (ANAs), anti-neutrophil cytoplasmic antibodies (ANCAs), and rheumatoid factor in sera from the index patients were performed by the clinical diagnostic laboratory of Sohag Medical Center, Sohag, Egypt, using direct immunofluorescence.

### Data availability.

All microarray data were deposited in the NCBI’s Gene Expression Omnibus database (GEO GSE186391).

### Statistics.

Data are expressed as means ± SEM unless stated otherwise. Experiments were performed in 3–4 biological replicates, and data were analyzed using GraphPad Prism 5.0 (GraphPad Software Inc.). Significance of between-group differences was assessed using Student’s *t* test (applying Bonferroni’s correction as indicated) and 1-way ANOVA followed by Tukey’s or Dunnett’s multiple comparisons. *P* values less than 0.05 were considered significant, using the following symbols: *P < 0.05, **P < 0.01, and ***P < 0.001. GO term enrichment analyses were performed with Metascape (http://metascape.org).

### Study approval.

The study falls in a category that is exempt from full ethics review (approval) owing to the use of existing, anonymized patient samples and low risk to the participants (State Board of Physicians, Lower Saxony, Germany). The parents gave informed consent, and the patients their assent, for use of data, images, and biosamples for the study.

## Author contributions

MNHM, SFHW, JC, RG, JZ, BA, RO, SM, MM, GG, LMR, SS, and TW performed the research. AME, ZAEG, and ARR treated the patients and provided clinical data. MS and PB performed the histological readings. UM and FM provided resources and supervision. All authors participated in interpreting the data and editing the manuscript. MNHM wrote the first draft of the manuscript. FP oversaw the project and wrote the final version of the manuscript.

## Supplementary Material

Supplemental data

## Figures and Tables

**Table 1:**
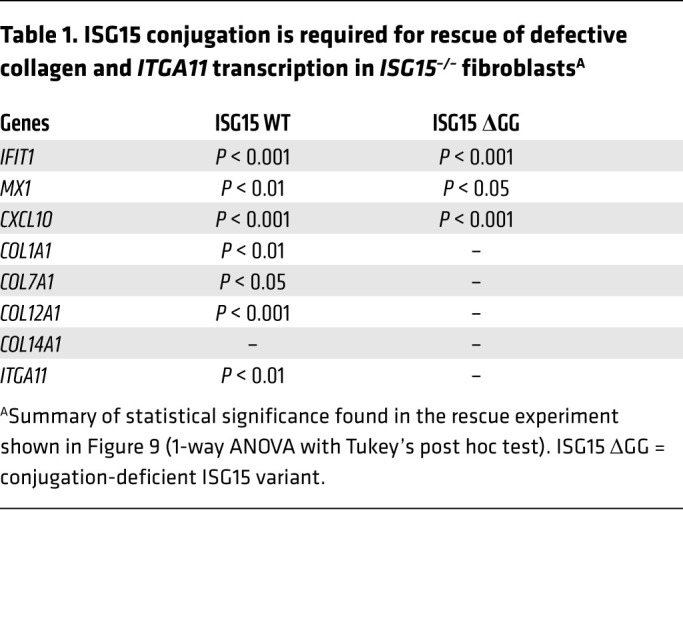
ISG15 conjugation is required for rescue of defective collagen and *ITGA11* transcription in *ISG15^–/–^* fibroblasts^A^

## Version 1 11/30/2021

In-Press Preview

## Version 2 02/01/2022

Electronic publication
