# Supplementary material for: Congenital deficiency reveals critical role of ISG15 in skin homeostasis
Source: J Clin Invest. 2022 Feb 1;132(3):e141573. doi: 10.1172/JCI141573 (PMC8803340; doi:10.1172/JCI141573)
Supplement: Supplemental data [file jci-132-141573-s270.pdf]

Supplemental Methods

Genomic and genetic analyses. *Whole exome sequencing* (WES) of the family was performed on peripheral blood genomic DNA (gDNA). The concentration and quality of the purified gDNA were assessed with a Bioanalyzer 2100 (Agilent Technologies; Waldbronn, Germany). Fragmentation of 200 ng gDNA in 55 µL Tris-EDTA buffer in a microtube (Covaris) was performed on a Covaris S2 sonication device (duty cycle 10%, intensity 4, 200 cycles per burst during 80s) to obtain fragments with an average length of 300 base pairs (bp). The size of the fragments was checked with the Agilent Bioanalyzer. The DNA sequencing library was generated from 200 ng of fragmented gDNA using Agilent SureSelectXT Reagent Kits v5 + UTRs (75 Mb) according to the manufacturer´s protocols. The final DNA sequencing library was purified, checked for size on the Agilent Bioanalyzer (High Sensitivity DNA Chip), and prepared for sequencing according to the manufacturer´s protocol (Illumina). The libraries were sequenced on an Illumina HiSeq2500 using TruSeq SBS Kit v3-HS (200 cycles, paired end run) with an average of 12.5 $x10^6$ reads per single exome (mean coverage: 50X). The quality of FASTQ files was determined before and after trimming with the FastQC tool. Raw reads were further manipulated by TrimGalore! (default settings) in order to clip Illumina adapter sequences and to clip/remove poor quality sequence reads. Trimmed FASTQ files were aligned to human reference genome hg19 using the BWA aligner tool followed by tagging duplicated reads (PCR products) with MarkDuplicates (Picard tools). Resulting BAM files are ready to use for SNV calling. Further quality measures (i.e. coverage and uniformity of exome data) were assessed by R package TEQC.

Single nucleotide variation (SNV) analysis. SNV calling was done using The Genome Analysis Toolkit (GATK) v3.3 and according to recommendations published under "Best Practices:

Germline short variant discovery (SNPs + Indels)” (https://gatk.broadinstitute.org/hc/en-us). Briefly, BAM files were passed through a pipeline consisting of modules “HaplotypeCaller (GVCF mode), GenotypeGVCF, and VariantRecalibrator. Refined genotypes were annotated using SnpEff 4.1 and dbNSFP v3.1.

Copy number variation (CNV) analysis was performed using CNVkit with default settings. HMZDelFinder (1) was applied to identify rare and intragenic homozygous and hemizygous (HMZ) deletions.

Short tandem repeat (STR) analysis was performed using the A20100 Human STR Primer Set kit (Sciex) according to the manufacturer’s recommendations.

Mass spectrometry (MS)-based proteomics. Cell pellets of IFN-α treated WT and *ISG15*$^{-/-}$ fibroblasts were resuspended in 50 μL of digestion buffer containing 4% SDS in 100 mM Tris (pH 7.6) and sonicated with a Bioruptor (Diagenode, Liege, Belgium) for 15 min at 4°C. Proteins were reduced with 10 mM dithiothreitol for 15 min at 90°C and for another 10 min at RT. Subsequent alkylation with 55 mM iodoacetamide was performed in the dark for 20 min at RT. Proteins were precipitated with acetone overnight at -20°C. Cell pellets were air dried and re-solubilized in 45 mL 10 mM HEPES containing 8 M urea. After sonication, proteins were digested overnight at 37°C with 0.5 µg Lys-C and 0.5 µg Trypsin, and digestion was then stopped with trifluoroacetic acid. Peptides were desalted using two discs of C18 material and resuspended in 10 μL of solution A (2% acetonitrile, 0.1% trifuoroacetic acid) for LC-MS. Peptides were separated on an EASY-nLC™ 1200 HPLC system (Thermo Fisher Scientific) via in-house packed columns (75-µm inner diameter, 30-cm length, and 1.9-µm C18). A buffer system was used consisting of buffer A (0.5% formic acid) and buffer B (0.5% formic acid and 80% acetonitrile) with a linear gradient of 5% to 30% B in 95 min at 300 nL/min flow. The column temperature was set to 60°C. A

Quadrupole Orbitrap mass spectrometer (Q Exactive HF, Thermo Fisher Scientific) was coupled to the LC via a nano-electrospray ion source. The mass spectrometer was operated in a data-dependent mode, collecting MS1 spectra (60,000 resolution, 300 to 1650 m/z scan range) with an automatic gain control and a maximum injection time of 20 ms. Up to the 15 most abundant ions from the MS1 scan were isolated with an isolation window of 1.4 m/z. Following higher-energy collisional dissociation with a normalized collision energy of 27%, MS2 spectra were collected (15,000 resolution, 200 to 2000 m/z scan range) with a maximum injection time of 28 ms. Dynamic precursor exclusion was set to 30 s to minimize resequencing. Data were acquired using Xcalibur software (Thermo Fisher Scientific).

MS data analysis and statistics. Mass spectra were searched against the 2018 UniProt human databases using MaxQuant (1.6.5.0) with a 1% false discovery rate (FDR) at the peptide and protein level. Peptides required a minimum length of 7 amino acids with carbamidomethylation as a fixed modification and with N-terminal acetylation and methionine oxidations as variable modifications. Enzyme specificity was set C-terminal to arginine and lysine using trypsin as the protease, and allowing a maximum of two missed cleavages in the database search. The maximum mass tolerances for precursor and fragment ions were 4.5 ppm and 20 ppm, respectively. Match between runs was enabled to transfer peptide identifications between individual measurements with a match time window of 0.7 min. Label-free quantification was performed with the MaxLFQ algorithm using a minimum ratio of 2.0. Protein identifications were filtered by removing matches to the reverse database, matches only identified by site, and common contaminants. Data were further filtered such that only proteins with identifications in at least 3 replicates of one condition were retained. Missing values were imputed from a normal distribution (width = 0.3, shift = 1.8). For pairwise comparison of proteomes and determination

of significant differences in protein abundances, t test statistics were applied with a permutation-based FDR of 5% and S0 of 1. All annotations were extracted from UniProtKB. Statistical analyses were performed with R Studio v3.4.4.

Amino acid measurements by mass spectrometry. The simultaneous absolute quantification of glycine, proline, and 4OH-proline were accomplished by liquid chromatography (Agilent 1290 Infinity II LC, Santa Clara, CA, USA) and mass spectrometry (AB SCIEX 5500 QTrap™ mass spectrometer; AB SCIEX, Darmstadt, Germany) using the AbsoluteIDQ® p180 kit (Biocrates, Life Science AG, Innsbruck, Austria). Extraction of metabolites and all analytical assays were performed according to the manufacturer's recommendations (UM_p180_ABSciex_11 and Application Note 1003-1, Biocrates Life Science AG, Innsbruck, Austria) and as described in (2).

RT-qPCR analysis. RNA was extracted using the Nucleospin RNA purification kit (Macherey-Nagel, Düren, Germany) and reverse-transcribed with the PrimeScript™ kit (Takara, Shiga, Japan) according to the manufacturers' protocols. Levels of mRNAs encoding various genes were analyzed using the SensiFast™ SYBR® No-ROX Kit (Bioline, Taunton, MA, USA) and a LightCycler480 instrument (Roche, Mannheim, Germany). Hypoxanthine-guanine phosphoribosyltransferase (HPRT) mRNA was used as internal reference. Relative expression of mRNA targets was measured by the $2^{-\Delta\Delta CT}$ method (3). Primer sequences are listed in Table S7.

Western blotting. Cells were lysed in modified RIPA buffer and subjected to immunoblotting using the following antibodies: Human ISG15 (raised against aa 1-150; sc-166755, 1:500); TGF-β1 (sc-130348, 1:500) and STAT1 (1:500) (all Santa Cruz Biotechnology, Texas, USA); pSTAT1 (58D6, 1:1000); IFIT1 (14769S, 1:1000); MX1 (37849S, 1:1000); AKT (4691S, 1:1000); pAKT (4060S, 1:2000) (Cell Signaling Technology, Massachusetts, USA); MMP1 (MAB901, 1:200) (R & D Systems, Minneapolis, USA); β-Actin (ab49900, 1:20,000) (Abcam, Cambridge, UK); TIMP1

(MA-13688, 1:200), and ALDH2 (MA5-17029, 1:1000) (Thermo Fisher). For detection, membranes were incubated with Amersham enhanced chemiluminescence western blot detection reagent (GE Healthcare Science, Pittsburgh, USA) and relative band intensities were determined using an iNTAS imaging device (iNTAS Science Imaging, Göttingen, Germany).

Transmission electron microscopy (EM). Paraffin-embedded samples were re-embedded for TEM modified from a protocol described in (4). On day 1, surplus paraffin was removed with a scalpel and the remaining block (approx. 4x4 mm) dewaxed twice in 2 mL xylene at 60°C in a heating block for 30 min each, followed by another xylene step overnight. On day 2, samples were rehydrated (5 min xylene-EtOH [1:1], 5 min 100% EtOH, 2 x 10 min 90% EtOH, 2 x 10 min 70% EtOH, 2 x 10 min 50% EtOH) and washed in 0.1 M EM-HEPES buffer (0.1 M HEPES, 0.09 M sucrose, 10 mM CaCl2, 10 mM MgCl2, pH 6.9). The samples were treated with 1% OsO4 (in EM HEPES buffer) for 1 h at RT, washed twice with 0.1 M EM-HEPES buffer and dehydrated in a graded serious of ethanol on ice (10%, 30%, 50%, 70%, 90% and 2x 100% EtOH for 15 min each). The dehydrated blocks were infiltrated with LR White (LRW) resin: 1:1 LRW-EtOH (overnight), 2:1 (24 h), 2x 100% LRW (each 24 h). Samples were polymerized at 75°C for 15 h and the block trimmed to enable orthogonal sectioning of the cell layers (Leica Ultratrim and Ultramicrotome Ultracut S; Leica, Wetzlar, Germany). Ultrathin sections of approx. 50-70 nm thickness were counterstained with 4% aqueous uranyl acetate for 3 min and lead citrate for 30 seconds. Images were acquired with a Libra 120 Plus (Zeiss, Germany) with an acceleration voltage of 120 kV at calibrated magnifications. Contrast and brightness adjustments as well as size measurements were done with the WinTEM software. Desmosomes were counted manually according to their characteristic appearance by two different examiners on two biological replicates. For Figure 7D, we evaluated 80 images at 4000x magnification (38 μm^2 per image).

References (Supplemental Methods)

1. Bigio B, Seeleuthner Y, Kerner G, Migaud M, Rosain J, Boisson B, et al. Detection of homozygous and hemizygous complete or partial exon deletions by whole-exome sequencing. *NAR Genom Bioinform.* 2021;3(2):lqab037.
2. Arshad H, Alfonso JCL, Franke R, Michaelis K, Araujo L, Habib A, et al. Decreased plasma phospholipid concentrations and increased acid sphingomyelinase activity are accurate biomarkers for community-acquired pneumonia. *J Transl Med.* 2019;17(1):365.
3. Livak KJ, and Schmittgen TD. Analysis of relative gene expression data using real-time quantitative PCR and the 2(-Delta Delta C(T)) Method. *Methods.* 2001;25(4):402-8.
4. Lighezan R, Baderca F, Alexa A, Iacovliev M, Bonte D, Murarescu ED, et al. The value of the reprocessing method of paraffin-embedded biopsies for transmission electron microscopy. *Rom J Morphol Embryol.* 2009;50(4):613-7.

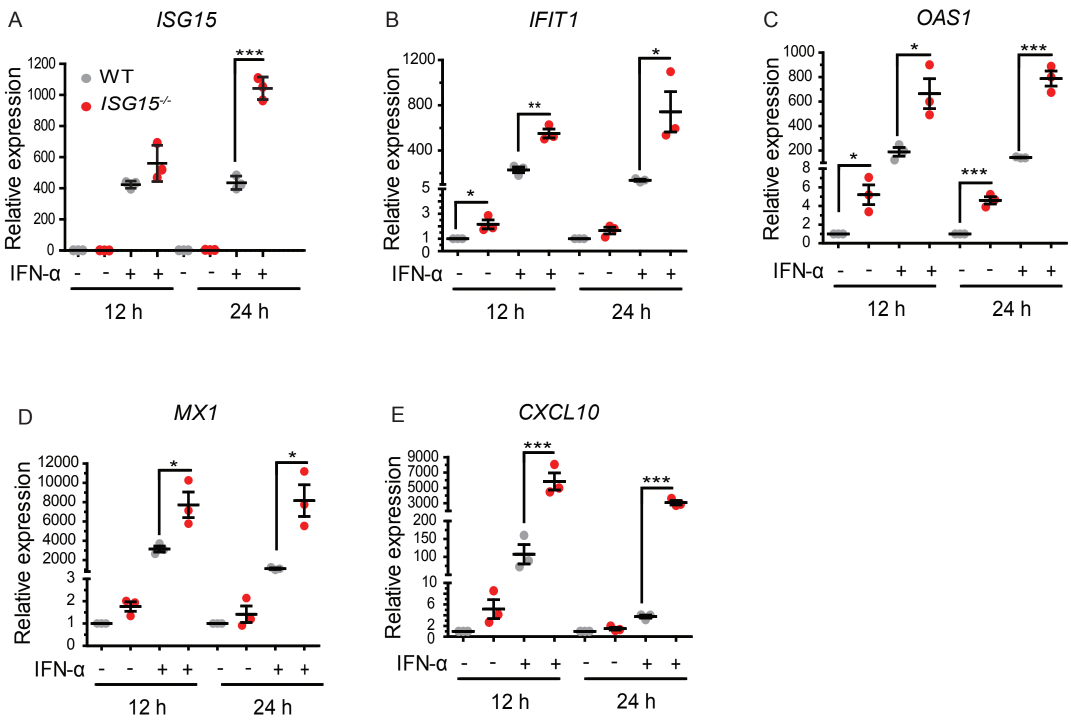

Figure S1. Hyperinflammation in IFNα stimulated *ISG15*$^{-/-}$ fibroblasts. WT or *ISG15*$^{-/-}$ hTERT-immortalized fibroblasts were stimulated with IFNα (1000 IU/mL) for the indicated times and analyzed by RT-qPCR for the five targets indicated in the graphs. ΔΔCt method, expression in unstimulated WT cells used as reference. **A.** ISG15 mRNA is strongly induced in WT and *ISG15*$^{-/-}$ cells (ISG15 protein is absent in these *ISG15*$^{-/-}$ cells, likely due to instability (23)). **B-E.** There is markedly higher induction of *IFIT1*, *OAS1*, *MX1,* and *CXCL10* mRNA in the *ISG15*$^{-/-}$ cells. n = 3 ±SEM; p * = <0.05, ** = <0.01, *** = <0.001 (Student's t-test).

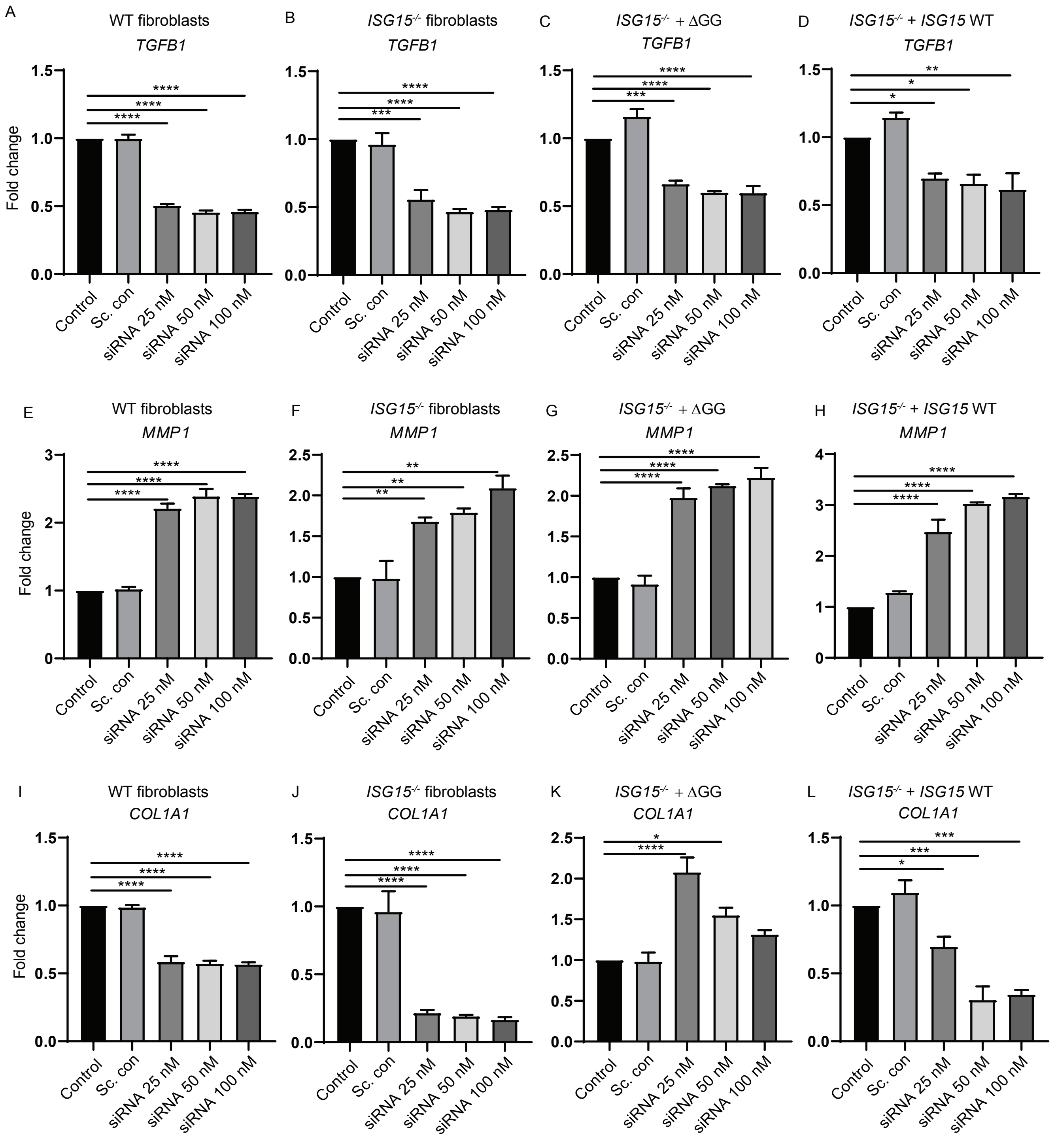

Figure S2. Knocking down *TGFβ1* expression increases *MMP1* and decreases *COL1A1* mRNA expression. *TGFβ1* mRNA was knocked down with siRNA in WT or *ISG15*$^{-/-}$ fibroblasts, and in *ISG15*$^{-/-}$ fibroblasts stably expressing WT ISG15 or the conjugation-deficient ΔGG variant. Expression of *MMP1* and *COL1A1* mRNA was measured by RT-qPCR 48 h after IFNα-stimulation. **A-D.** An approx. 50% reduction is achieved in the four cell variants. **E-H.** Conjugation-independent increase in *MMP1* mRNA expression. **I-L.** Conjugation-independent decrease in *COL1A1* expression. n = 3 ± SEM; p * <0.05, ** <0.01, *** <0.001 (one-way ANOVA with Tukey's post-hoc test).

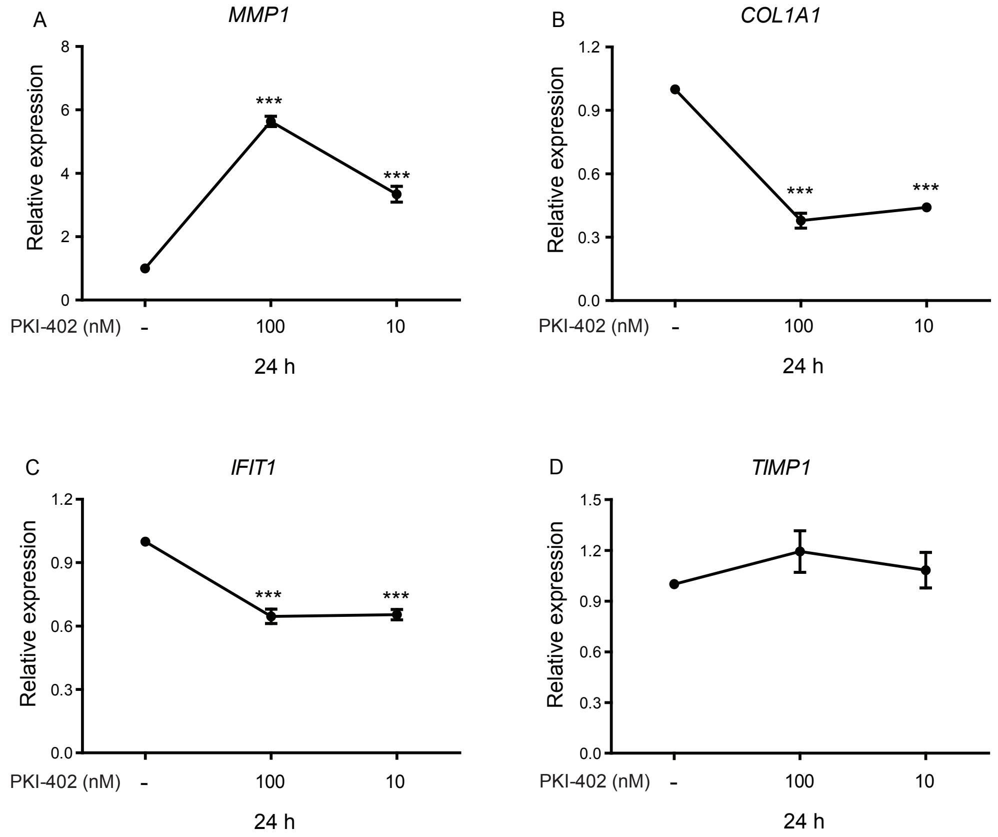

Figure S3. PI3K inhibition in immortalized WT fibroblasts for 24 h phenocopies the effect of ISG15 deficiency on *COL1A1* and *MMP1* expression. Cells were treated with 10 or 100 nM PKI-402 for 24 h and analyzed by RT-qPCR for expression of the four targets indicated in the graphs. $\Delta\Delta$Ct method, expression in unstimulated WT cells used as reference. **A-B.** Reduction of *COL1A1* and induction of *MMP1*. **C.** Downregulation of *IFIT1*. **D.** As opposed to the reduction seen after 3 h of treatment (Figure 4), *TIMP1* expression is unchanged. n = 3 ±SEM; p * <0.05, ** <0.01, *** <0.001 (one-way ANOVA with Dunnett's post-hoc test).

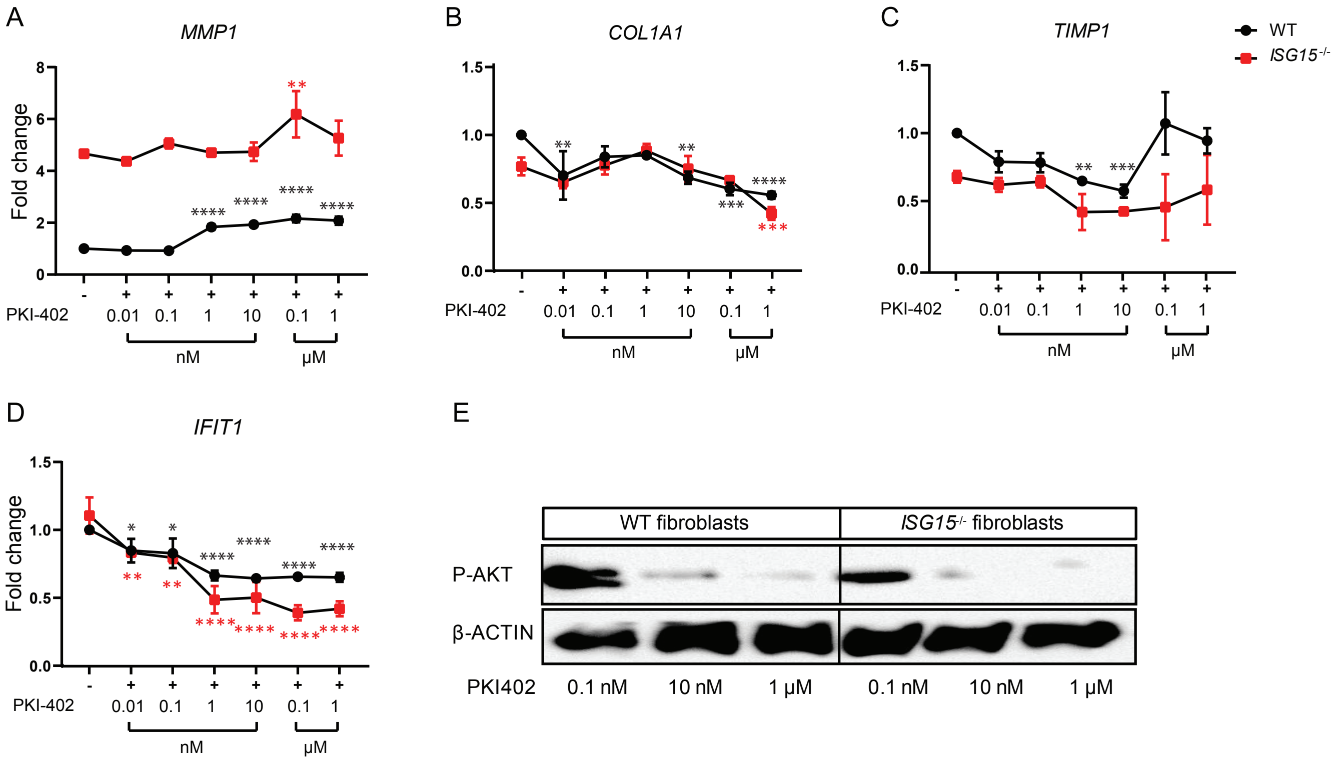

Figure S4. PI3K inhibition reduces AKT phosphorylation and *COL1A1* and *IFIT1* mRNA expression in WT and *ISG15*$^{-/-}$ immortalized fibroblasts. Cells were incubated in the presence of the indicated concentrations of PKI-402 for 3 h. **A-D,** Expression of the indicated mRNA targets (RT-qPCR). *n* = 3 ±SEM; *p* * = <0.05, ** = <0.01, *** = <0.001 (One way ANOVA with Dunnett's post-hoc test) **E,** AKT phosphorylation is lower in *ISG15*$^{-/-}$ cells and decreases in both genotypes in the presence of PKI-402 in a dose-dependent manner (anti-P-AKT immunoblot).

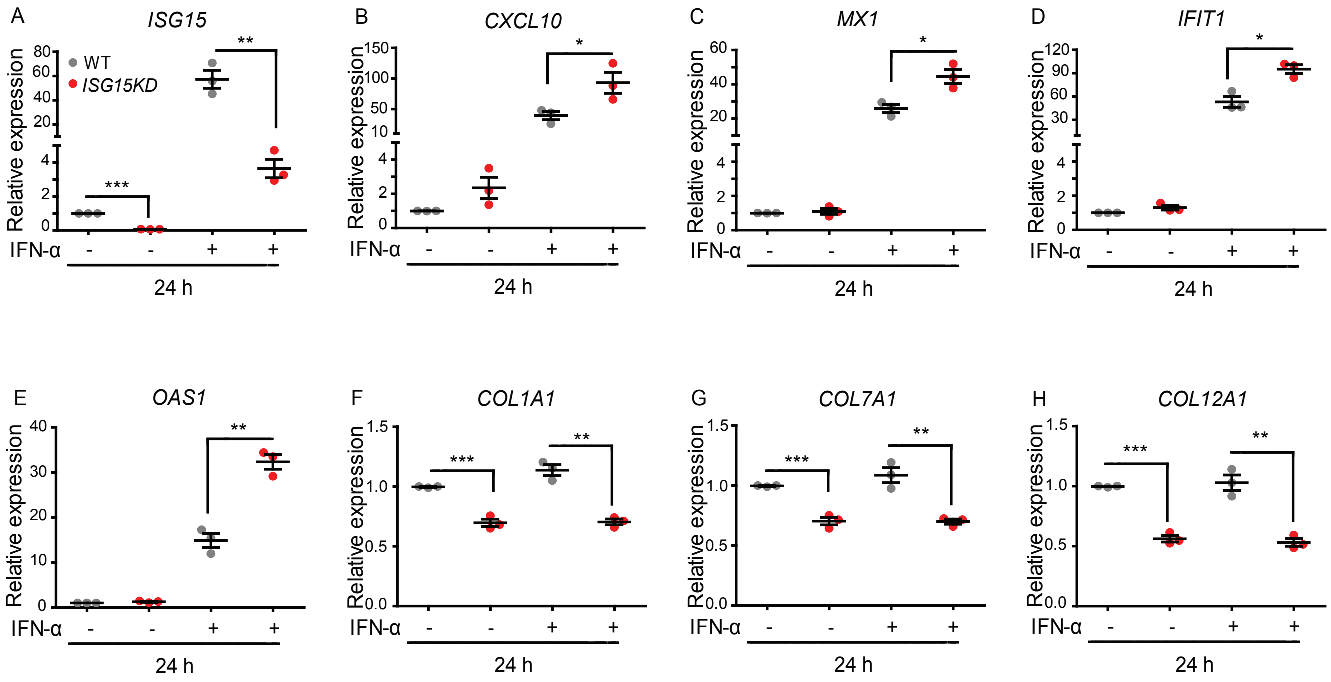

Figure S5. Hyperinflammation and reduced collagen synthesis after knock-down of *ISG15* in primary keratinocytes. Cells were stimulated with IFNα (1000 IU/mL) for 24 h and analyzed by RT-qPCR for expression of the 8 targets indicated in the graphs. ΔΔCt method, expression in unstimulated WT cells used as reference. **A.** Strong knock-down of *ISG15* mRNA expression with siRNA. Significantly higher levels of all inflammatory markers are seen after IFNα stimulation **(B-E),** whereas lower collagen expression is also evident in the unstimulated knock-down cells (labeled $ISG15^{-/-}$) **(F-H)**. *n* = 3 ±SEM; *p* * = <0.05, ** = <0.01, *** = <0.001 (Students's *t*-test).

A

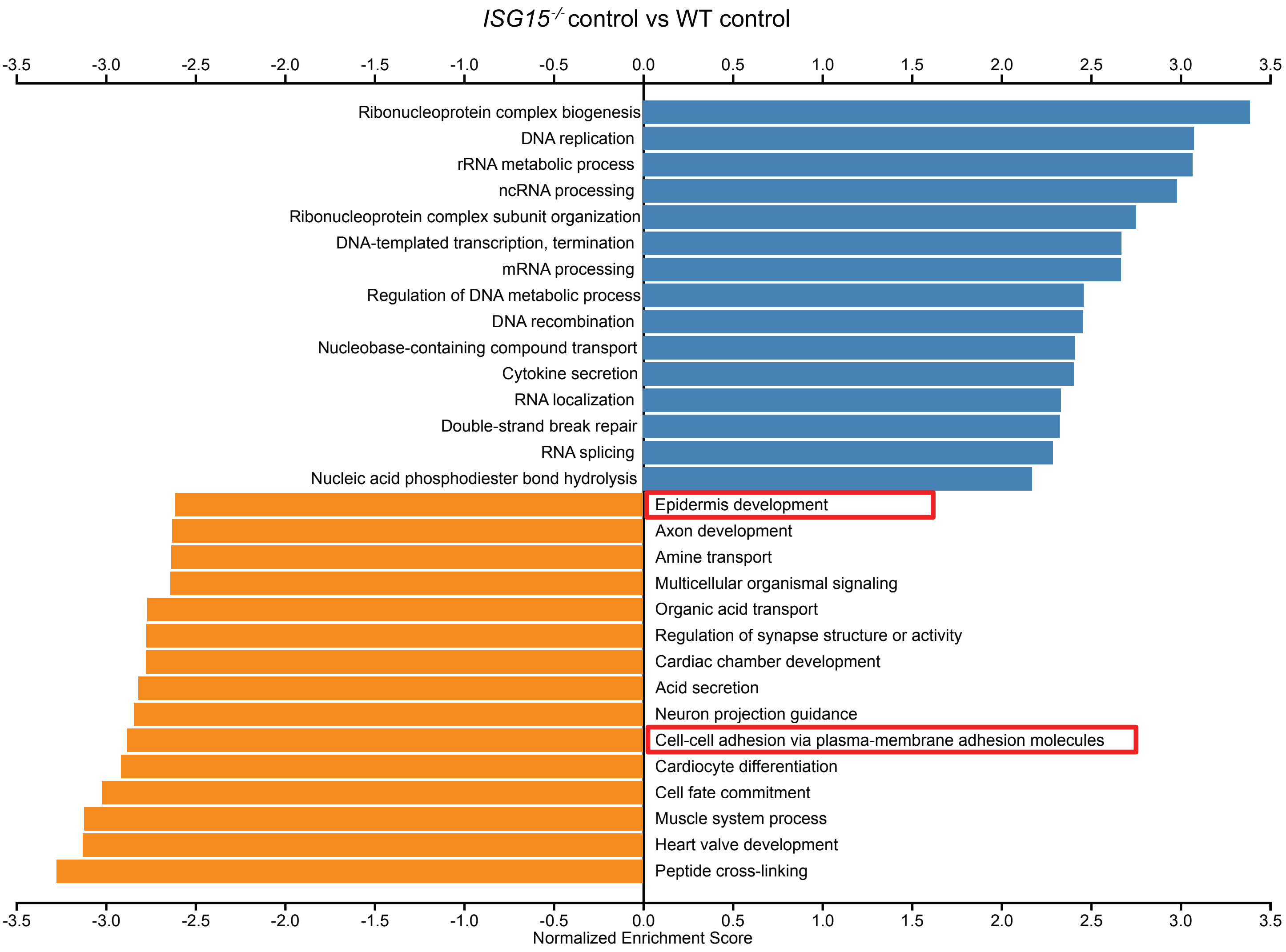

Figure S6. Dysregulation of pathways relating to cell differentiation and epidermis development in *ISG15*$^{-/-}$ HaCaT keratinocytes. Gene expression was determined by microarray analysis in unstimulated WT and *ISG15*$^{-/-}$ HaCaT cells and after 24 h of stimulation with IFNα (1000 IU/mL). **A.** Gene ontology (GO) enrichment analysis comparing unstimulated WT and *ISG15*$^{-/-}$ cells. **B.** Differentially expressed (p adjusted <0.05, Student's *t*-test) targets in the data used for A. **C.** GO enrichment analysis comparing unstimulated WT and *ISG15*$^{-/-}$ cells. **D.** Differentially expressed (p adjusted <0.05, Student's *t*-test) targets in the data used for **C**. The color scale refers to fold change ($\log_2$). n = 3.

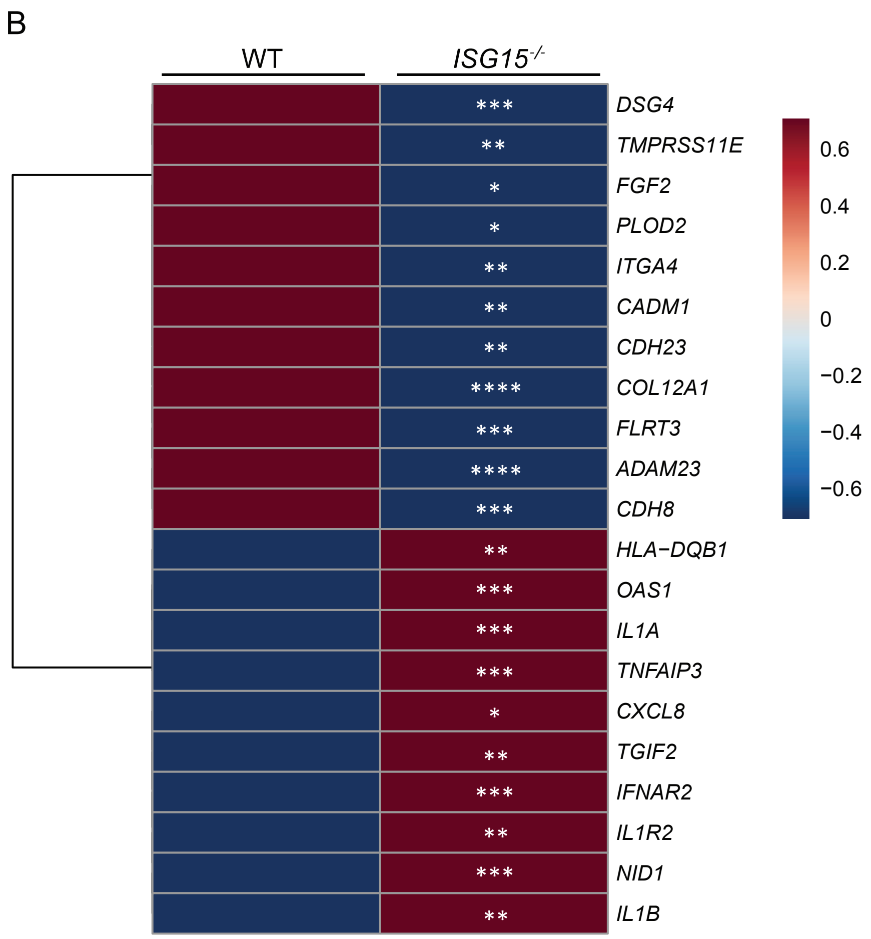

Figure S6B. Differentially expressed (*p* adjusted <0.05, Student's *t*-test) targets in the data used for A.

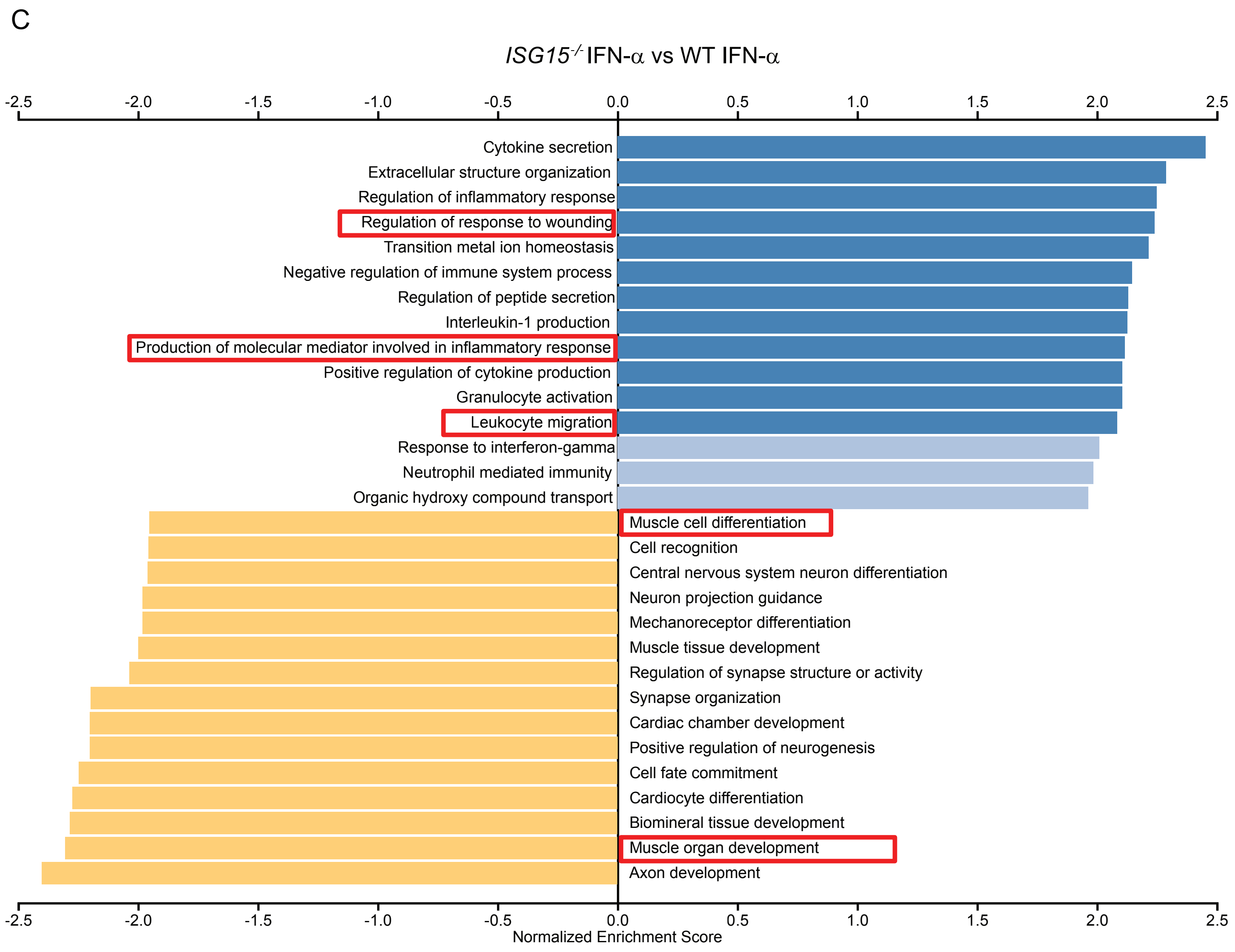

Figure S6C. GO enrichment analysis comparing unstimulated WT and *ISG15*$^{-/-}$ cells.

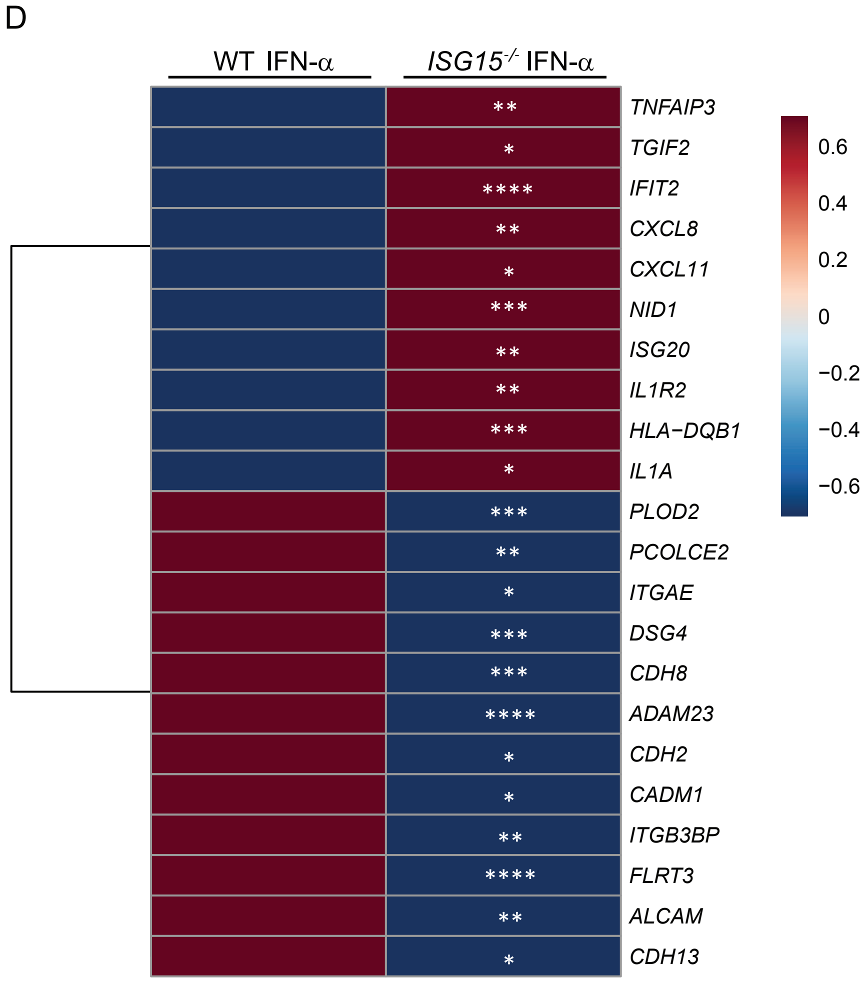

Figure S6D. Differentially expressed (p adjusted <0.05, Student's *t*-test) targets in the data used for **C**.

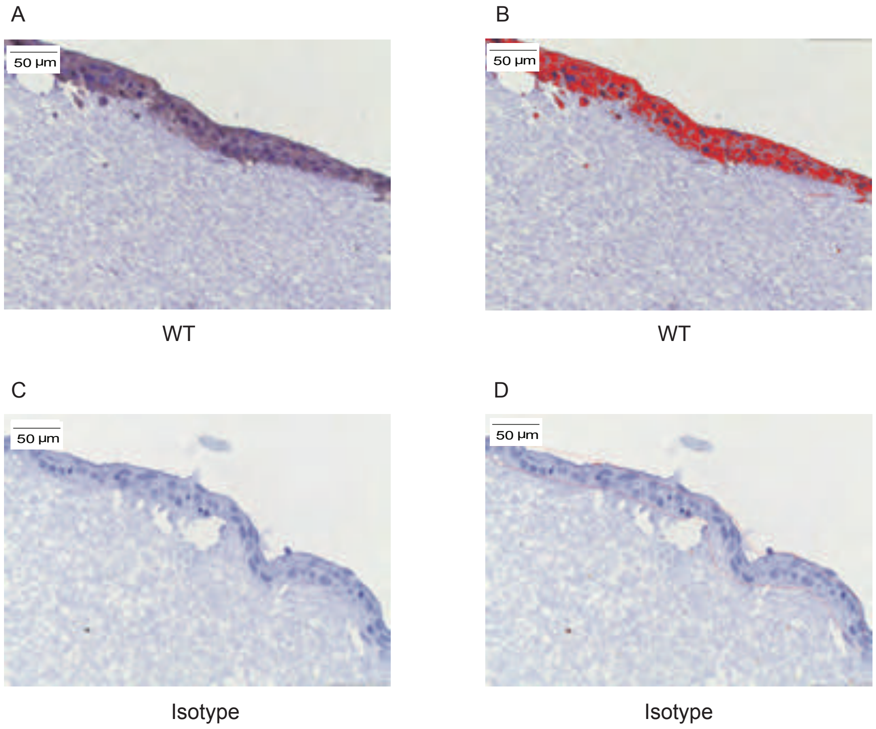

Figure S7. Absence of nonspecific signal in TGF-β1 immunostains of the 3D epidermis model. Paraffin-embedded tissue of an ISG15 WT model was immunostained by standard 3-step IHC using a specific anti-TGF-β1 antibody (**A,C**) or nonspecific mouse IgG1 at the same concentration as negative (isotype) control (**B,D**). DAB (brown) was used as chromogen. There is extensive intra- and extracellular expression of TGF-β1 expression when the specific antibody (**A**) but not when the control antibody (**C**) is used. The areas marked in red correspond to the TGF-β1 signal quantified by digital image analysis (cellSens Dimension software). This signal is seen only with the specific antibody (compare **B** vs. **D**).

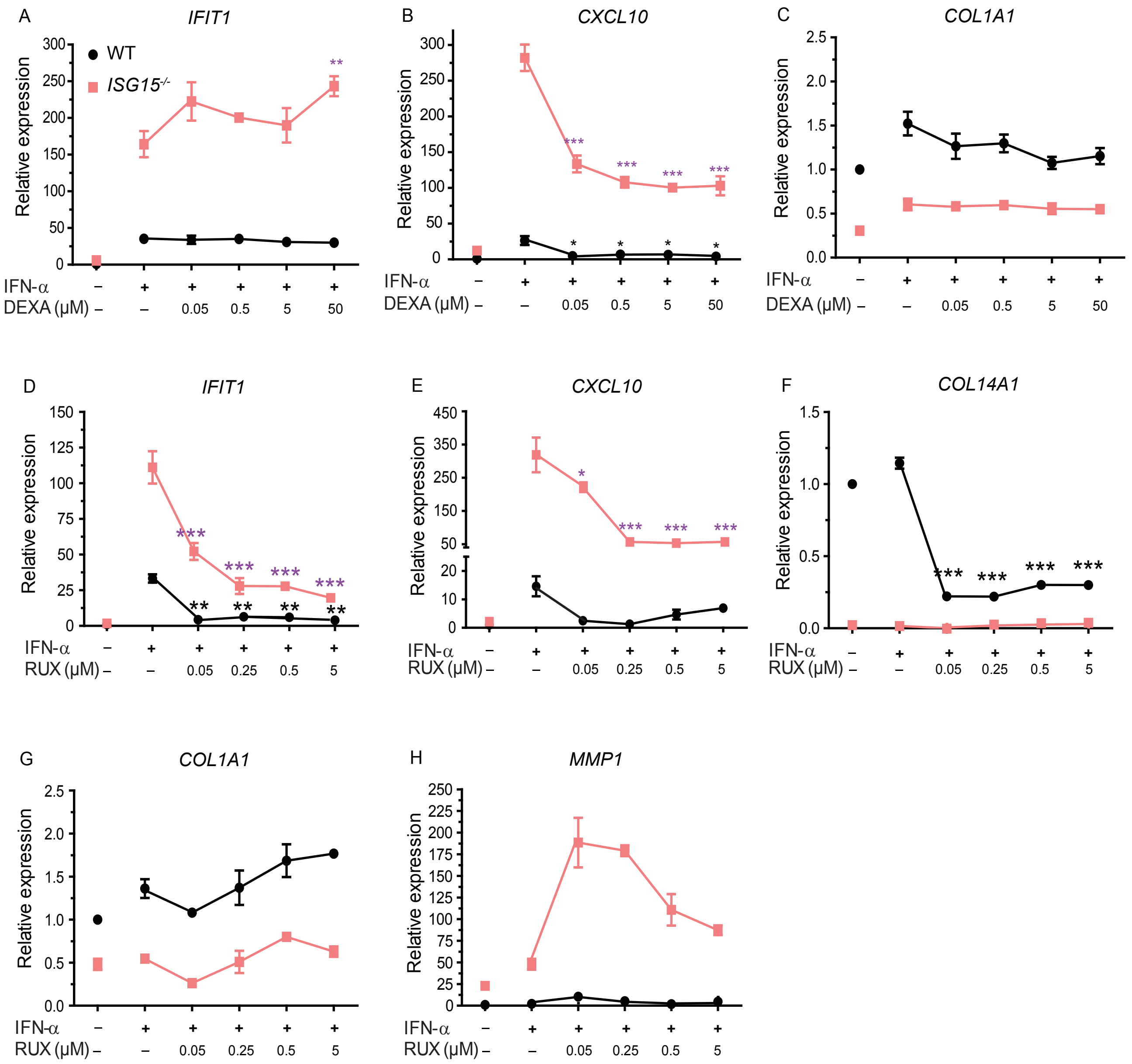

Figure S8. Effects of ruxolitinib and dexamethasone on WT and *ISG15*$^{-/-}$ fibroblasts. WT or *ISG15*$^{-/-}$ immortalized fibroblasts were stimulated with IFNα (1000 IU/mL) for 8 h and then treated for 16 h with increasing concentrations of ruxolitinib (RUX) or dexamethasone (DEXA). Expression of the mRNAs indicated in the graphs was measured by RT-qPCR. ΔΔCt method, expression in unstimulated WT cells used as reference. **A-C.** Treatment with dexamethasone. **E-H.** Treatment with ruxolitinib. An unexpected induction of *MMP1* mRNA by RUX is seen, which is inversely dose dependent (E). *n* = 3 ±SEM; *p* * = <0.05, ** = <0.01, *** = <0.001 (One way ANOVA with Tukey's post-hoc test).

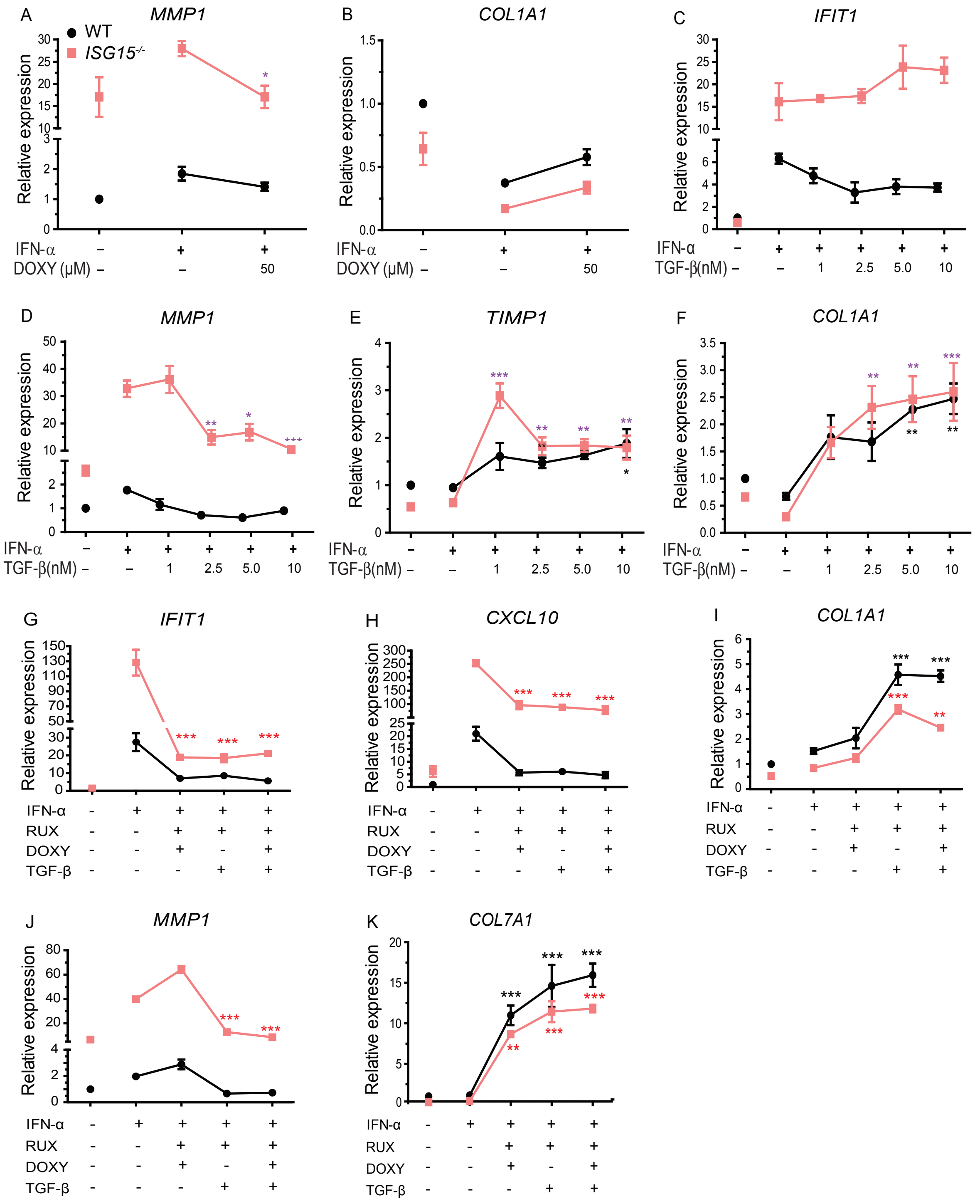

Figure S9. Effects of doxycycline, TGF-β1, and the combination of ruxolitinib/doxycycline/TGF-β1 on WT and *ISG15*$^{-/-}$ fibroblasts. WT or *ISG15*$^{-/-}$ immortalized fibroblasts were stimulated with IFNα (1000 IU/mL) for 8 h and then treated for 16 h with 50 μM doxycyline (DOX), increasing concentrations of TGF-β1, or ruxolitinib/doxycycline/TGF-β1. Expression of the mRNAs indicated in the graphs was measured by RT-qPCR. ΔΔCt method, expression in unstimulated WT cells used as reference. **A-B** Doxycycline. **C-F** TGF-β1. **G-K.** Combined treatment with ruxolitinib, dexamethasone, and TGF-β1. *n* = 3 ±SEM; *p* * = <0.05, ** = <0.01, *** = <0.001 (One way ANOVA with Tukey's post-hoc test).

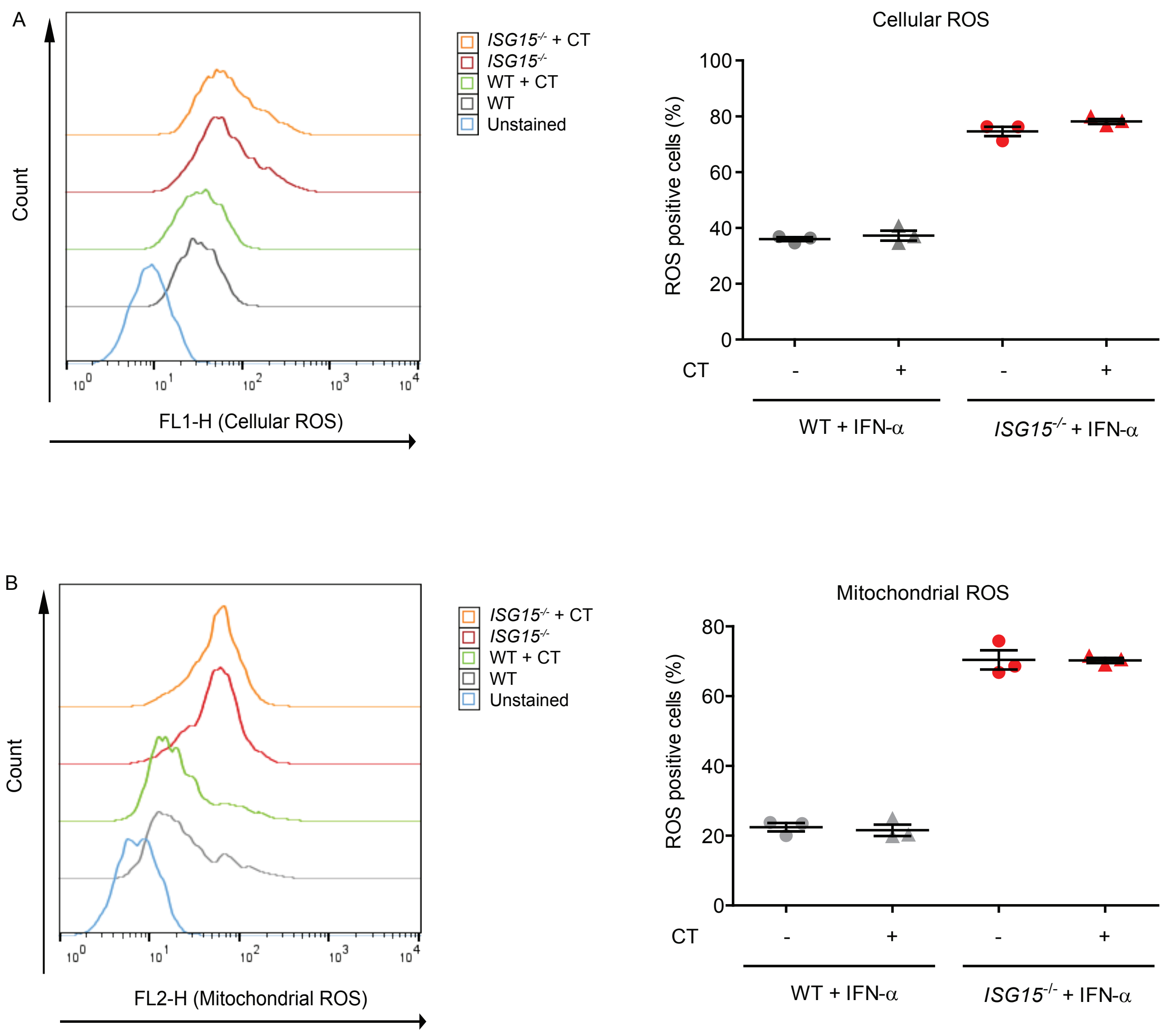

Figure S10. Treatment with the combination of ruxolitinib, doxycycline, and TGF-β does not normalize elevated ROS synthesis in *ISG15*$^{-/-}$ fibroblasts. WT or *ISG15*$^{-/-}$ immortalized fibroblasts were stimulated with IFNα (1000 IU/mL) for 8 h and then treated with a combination of ruxolitinib (0.5 μM), doxycycline (50 μM), and TGF-β1 (10 nM) for 16 h. Mitochondrial (**A**) and cellular (**B**) ROS were measured by flow cytometry. The treatment did not affect ROS levels. *n* = 3 ±SEM. CT = combined treatment.

A

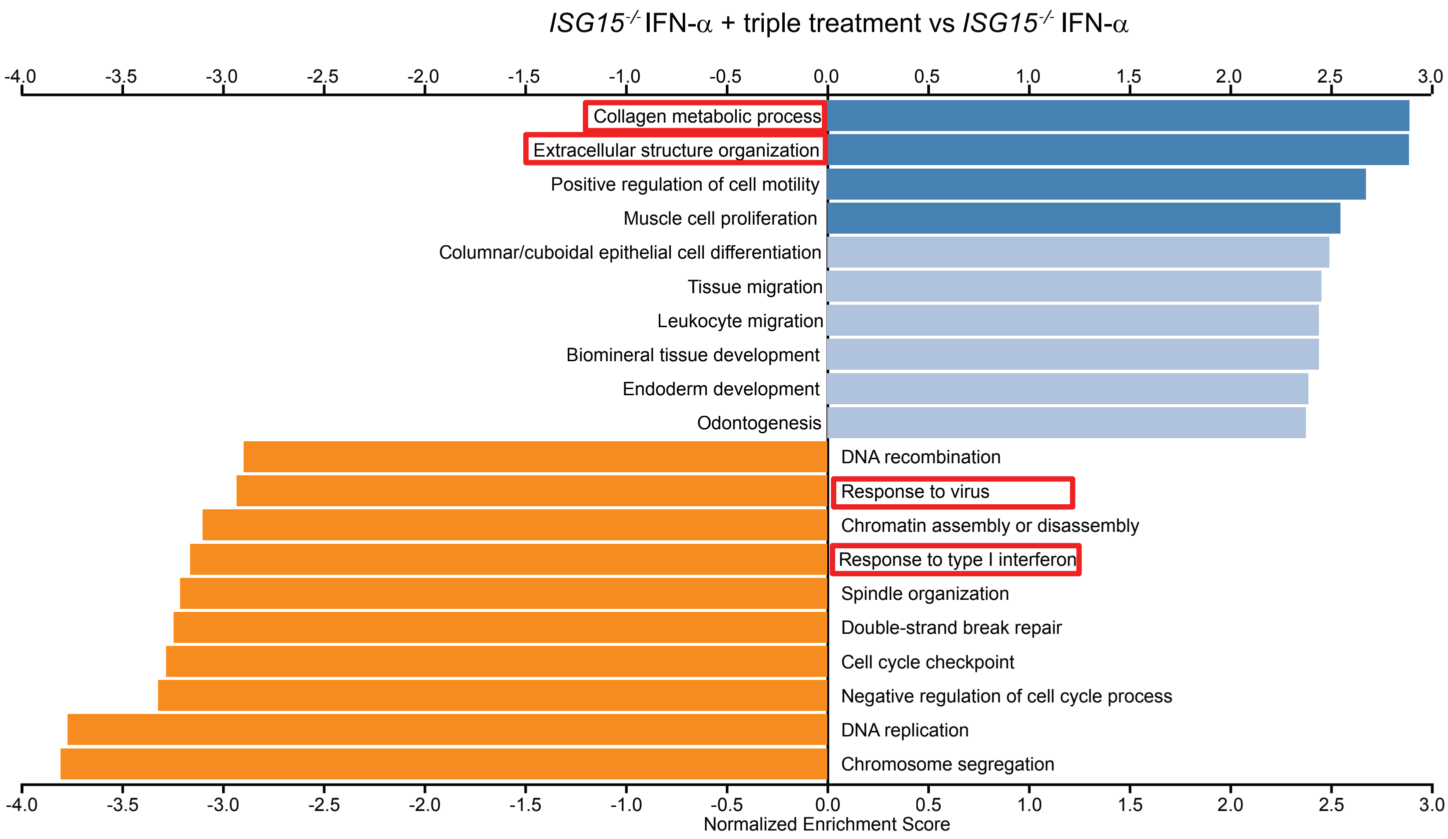

Figure S11. Treatment with the combination of ruxolitinib, doxycycline, and TGF-β activates collagen metabolism, extracellular matrix organization, and expression of the anti-inflammatory deubiquitinase TNFAIP3 in IFNα-stimulated *ISG15*$^{-/-}$ HaCaT keratinocytes. *ISG15*$^{-/-}$ HaCaT cells were stimulated with IFNα (1000 IU/mL) for 8 h and then treated with ruxolitinib (0.5 μM), doxycycline (50 μM), and TGF-β1 (10 nM) for 16 h. Gene expression was determined by microarray analysis. **A.** GO enrichment analysis comparing IFNα-stimulated treated vs. untreated cells. **B.** Expression of differentially expressed (p adjusted < 0.05) targets in the data used for **A**. *n* = 3.

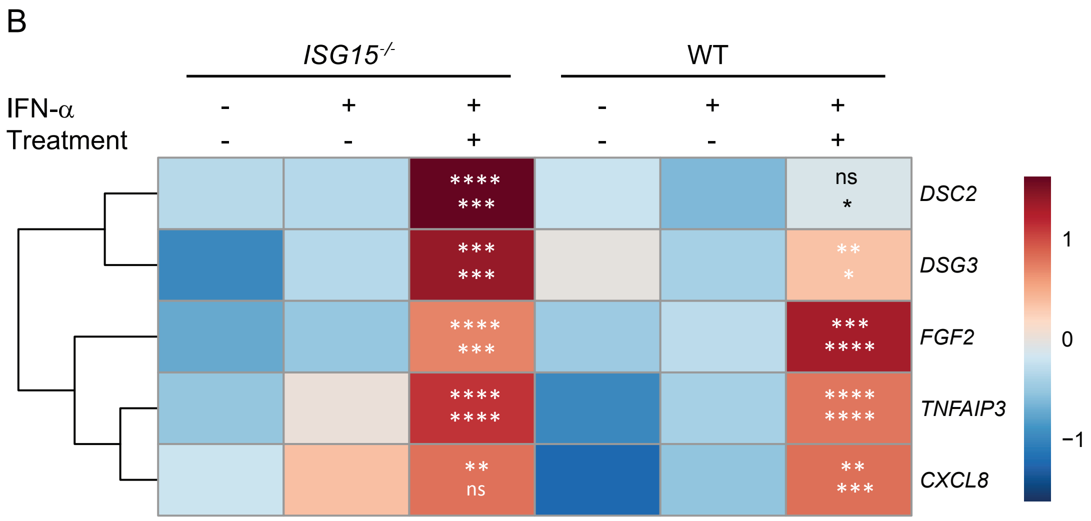

Figure S11B. Expression of differentially expressed (p adjusted < 0.05) targets in the data used for **A**.

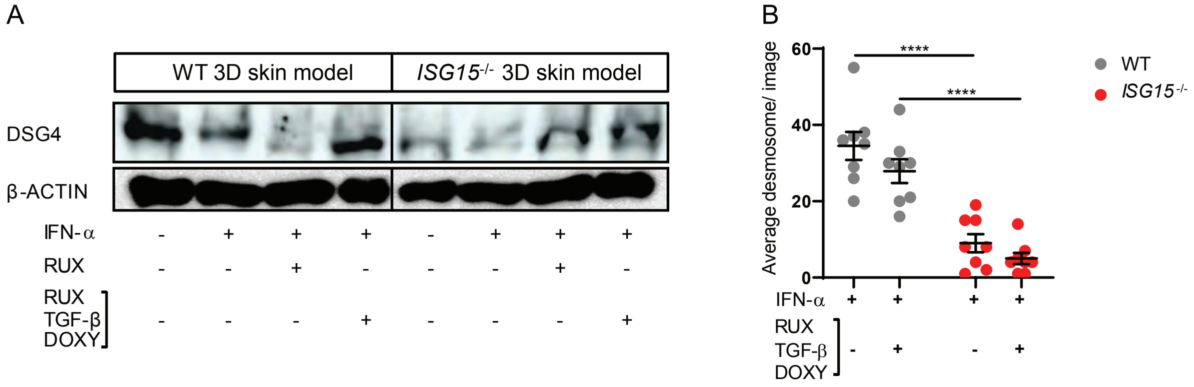

Figure S12. A. Treatment with ruxolitinib or ruxolitinib/doxycycline/TGF-β1 increases DSG4 expression in the *ISG15*$^{-/-}$ 3D epidermis model. WT and *ISG15*$^{-/-}$ 3D models were performed as before, subjected to RUX or triple treatment, and DSG4 expression was determined in lysates by immunoblot. **B. Treatment with RUX/DOXY/TGF-β1 does not increase desmosome density in the *ISG15*$^{-/-}$ 3D epidermis model.** Paraffin blocks of the experiment shown in Figure 11 were re-embedded for EM, and desmosome densities were determined by visual inspection as in Figure 7. Desmosome density is reduced in the *ISG15*$^{-/-}$ model, but does not increase upon triple treatment (n=8 images per group) n = 3 ± SEM; p * = <0.05, ** = <0.01, *** = <0.001 (One way ANOVA with Tukey's post-hoc test).

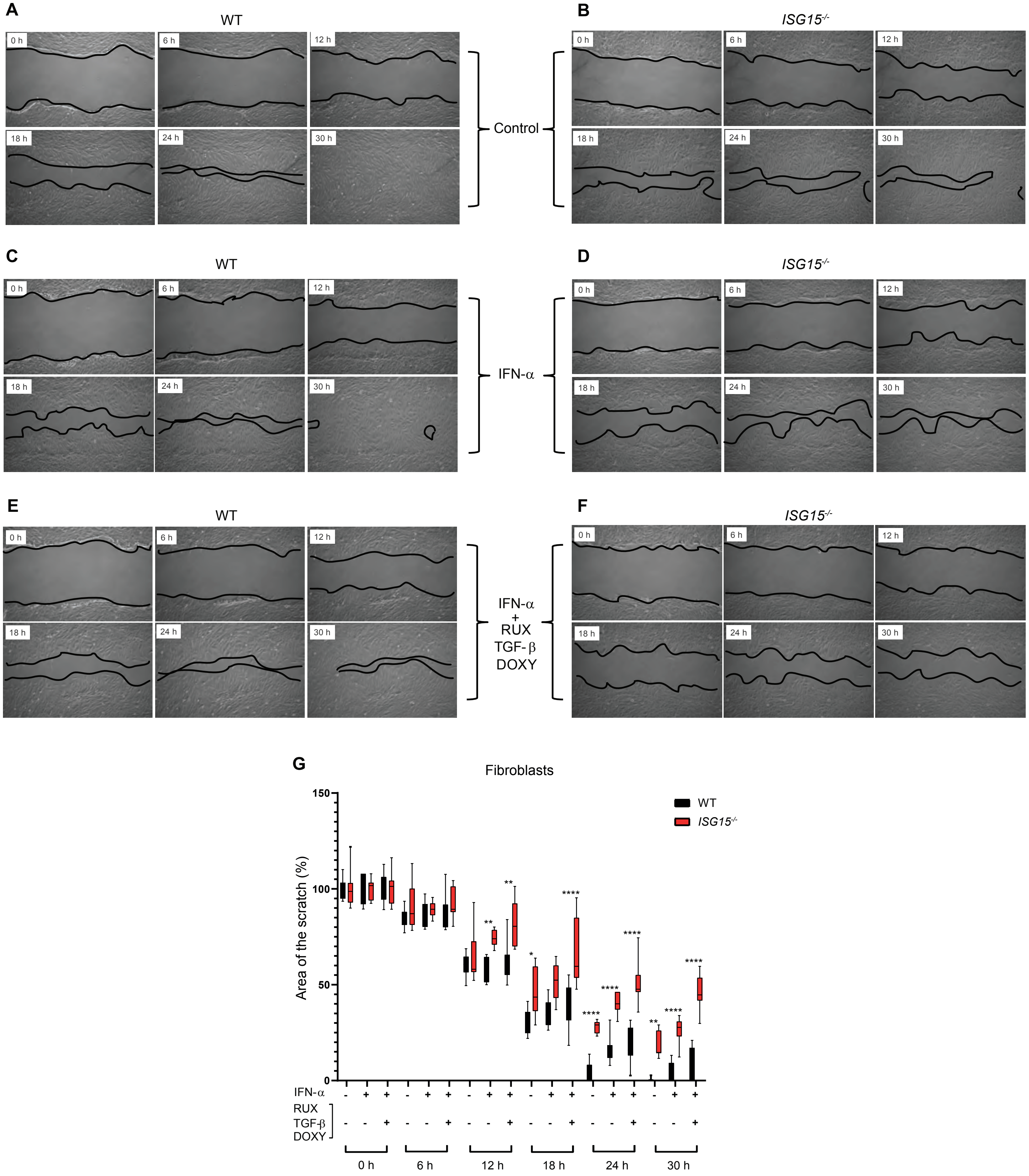

Figure S13. Cell migration is retarded in *ISG15*$^{-/-}$ immortalized fibroblasts and is delayed further by triple treatment with ruxolitinib/doxycycline/TGF-β1. Scratch assays were performed with WT or *ISG15*$^{-/-}$ HaCaT keratinocytes with or without triple treatment (RUX/DOXY/TGFβ) or single RUX or TGFβ treatment. Gap closure was measured by automated image analysis for the indicated time periods. Images show start and end. The black lines delineate the borders of the scratch. **A-C.** RUX/DOXY/TGF-β treatment. Gap closure is slower in *ISG15*$^{-/-}$ than WT cells and is retarded further by the treatment. Duration of experiment 60 h. n = 3 ±SEM; p * = <0.05, ** = <0.01, *** = <0.001 (one-way ANOVA with Tukey's post-hoc test).

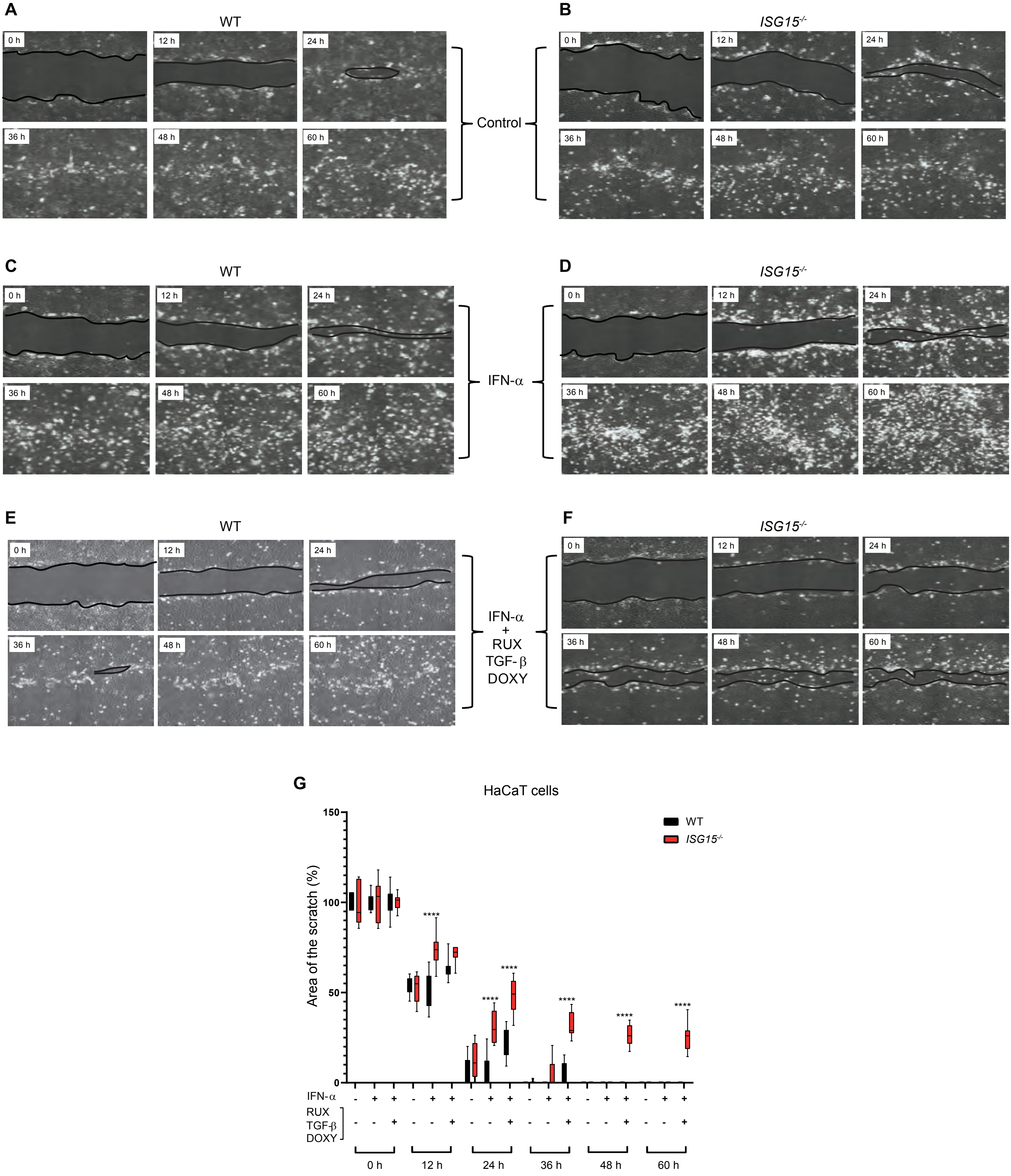

Figure S14. Cell migration is retarded in *ISG15*$^{-/-}$ HaCaT keratinocytes and is delayed further by triple treatment with ruxolitinib/doxycycline/TGF-β1. Scratch assays were performed with WT or *ISG15*$^{-/-}$ HaCaT keratinocytes with or without triple treatment (RUX/DOXY/TGFβ1) or single RUX or TGFβ1 treatment. Gap closure was measured by automated image analysis for 60 h. The black lines delineate the borders of the scratch. **A-C.** RUX/DOXY/TGF-β1 treatment. Gap closure is slower in *ISG15*$^{-/-}$ than WT cells and is retarded further by the treatment. *n* = 3, ±SEM; *p* * = <0.05, ** = <0.01, *** = <0.001 (one-way ANOVA with Tukey's post-hoc test).

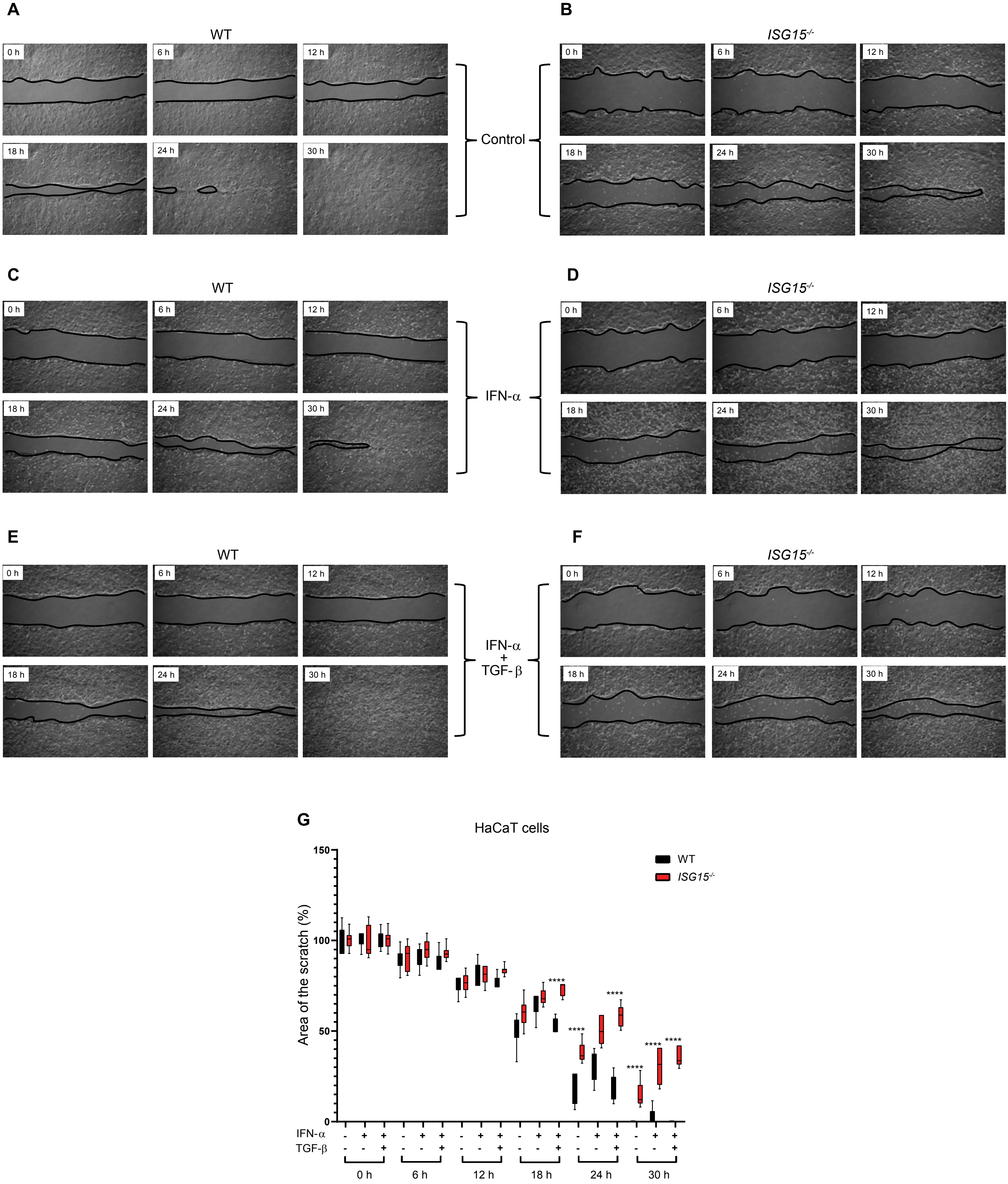

Figure S15. Cell migration is retarded in *ISG15*$^{-/-}$ HaCaT keratinocytes and is delayed further by treatment with TGF-β1. The figure shows images of all time points of the experiment shown in brief in Figure 12 A-B. *n* = 3, ± SEM; *p* * = <0.05, ** = <0.01, *** = <0.001 (one-way ANOVA with Tukey's post-hoc test).

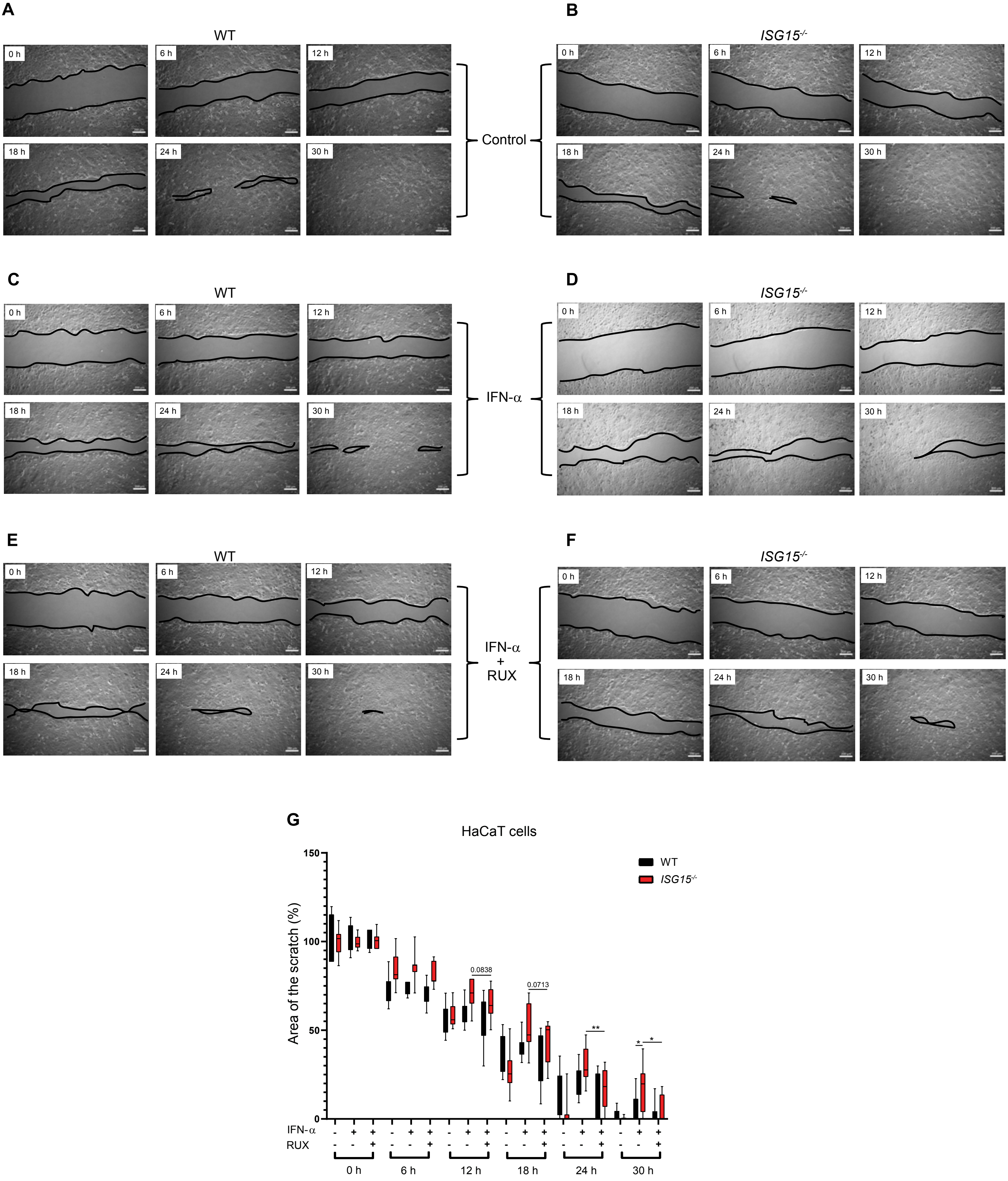

Figure S16. Treatment with ruxolitinib normalizes delayed cell migration in *ISG15*$^{-/-}$ HaCaT cells. The figure shows images of all time points of the experiment shown in brief in Figure 12 D,E. *n* = 3, ±SEM; *p* * = <0.05, ** = <0.01, *** = <0.001 (one-way ANOVA with Tukey's post-hoc test).

Table S1. Clinical parameters of autoimmunity and systemic inflammation

**Parameters**	**Patient (P1)**	**Patient (P2)**
ANA	Positive (1:40, homogeneous pattern)	Negative
P-ANCA	Negative	Negative
C-ANCA	Negative	Negative
Anti-GBM	Negative	Negative
ESR (after 1 h)	84 mm	20 mm
ESR (after 2 h)	106 mm	43 mm
CRP	24 mg/L (ref. <6 mg/L)	<6 mg/L

Table S2. Single nucleotide variant coverage, I*SG15* Y96*

**ID**	**Pedigree**	**Wild type/mutant**	**Coverage** [a]	**Genotype**
001	Father	C/G	37/0	C/C
002	Mother	C/G	54/52	C/G
003	Child (P1, m)	C/G	0/96	G/G
004	Child (P2, f)	C/G	0/117	G/G
005	Child	C/G	40/49	C/G
006	Child	C/G	39/41	C/G

[a] Next generation sequencing reads per allele

Table S3. Short tandem repeat analysis of the ISG15 deficiency kindred**.** The results are consistent with the pedigree shown in Figure 2.

Locus	001 Father	002 Mother	003* Case (m) P1	004* Case (f) P2	005 Sister	006 Brother
TH01	175,22		175,18	175,14	175,13	
	179,28	179,27	179,24	179,19	179,18	179,24
D18S51		303,66	303,68			303,66
		318,78		318,8	318,86	
	322,54			322,58	322,64	322,55
	337,67		337,7			
AMEL	104.91	104.88	104.90	104.84	104.91	104.89
	110.66		110.64			110.63
D13S317		189.42	189.41	189.35	189.39	189.40
	197.42		197.41	197.34	197.37	
	201.47					201.43
D7S820	226.03		225.98		226.05	225.95
	233.95	234.02	233.93	233.27	234.01	233.91
D16S539	284.64		284.56	284.55	284.57	
		288.64	288.61		288.62	288.56
	296.79	296.75		296.71		296.67
Penta E	398.11	398.09		398.04	398.08	398.06
		403.27	403.25			
	408.50		408.46		408.45	408.45
D3S1358	128.65			128.60	128.65	
	137.09	137.00	137.28	136.93	136.95	136.63
D8S1179		226.26		225.68	226.17	
	234.26	234.22	234.14		234.18	234.50
	238.23			237.65		
TPOX	272.09	272.06	272.01	271.55	272.16	271.97
CSF1PO	336.08	336.08	336.07			336.06
	344.14	344.12	344.12	343.67	344.18	344.11
Penta D		373.22	373.24		373.30	
	409.82		409.84	409.81	409.85	409.82
		424.32		424.31		424.34

Table S4. X chromosome haplotype analysis

ID	Pedigree	Haplotype 10.062.079	Haplotype 10.062.163
001	Father	T	G
002	Mother	T/A	G/C
003*	Child (P1, m)	A	C
004*	Child (P2, f)	T/T	G/G
005	Child (f)	T/A	G/C
006	Child (m)	A	C

Analysis based on the WES dataset, selecting a fragment containing two nearby sequence variants. The results exclude the possibility that the WES datasets of 001 and 006 were accidentally mixed up, as 006 cannot be the father of 004. *, index case.

**Table S5**. Nonsynonymous single nucleotide variation analysis of the ISG15 kindred. [a]			
**Amino acid change**	**Gene GenBank ID**	**Gene name**	**Relevant functional implication(s) of gene product** [b]
Y96*	ISG15 NM_005101.3	Interferon-stimulated gene 15	(this report)
Q97E	MXRA8 NM_001282585.1 Chr 1	Matrix remodeling associated 8	Cell adhesion, matrix remodeling (5)
D1363E	SPEN NM_015001.2 Chr 1	HDAC1-associated repressor protein (and others)	Epidermal sclerosis (*Drosophila*) (6)
R1917C	SPEN NM_015001.2	"-"	"-"
T312I	CLCNKA NM_004070.3	Chloride Voltage-Gated Channel Ka	--
R538C	CLCNKA NM_004070.3	"-"	--
D9E	GSTM1 NM_000561.3	Glutathione S-Transferase Mu 1	Psoriasis, atopic dermatitis, skin cancer (7)
S436G	FOXD1 NM_004472.2	Forkhead Box D1	Alleviates osteoarthritis (8)
P122S	SALL2 NM_005407.2	Spalt Like Transcription Factor 2	--
R209G	OR10G2 NM_001005466.2	Olfactory Receptor Family 10 Subfamily G Member 2	--
R187P	"-"	"-"	--
V118M	OR4E2 NM_001001912.2	Olfactory Receptor Family 4 Subfamily E Member 2	Psoriatic arthritis (9)
Q234R	"-"	"-"	"-"

[a] WES data were interrogated for nonsynonymous variants which are homozygous in the two index cases only. Additional filters were Mapping Quality > 60, dbNSFP_ExAC_Adj_AF (adjusted allele freq) < 0.05 or unknown, Nonsense or Missense Variant (no filter for nonsynonymous was applied).
[b] According to review of published literature.

Supplemental References (Table S5)

5. Walker MG, Volkmuth W. Cell adhesion and matrix remodeling genes identified by co-expression analysis. *Gene Funct Dis*. 2002;3(3–4):109–112.
6. Mace KA, et al. An epidermal barrier wound repair pathway in Drosophila is mediated by grainy head. *Science*. 2005;308(5720):381–385.
7. Cho HR, et al. Glutathione S-transferase M1 (GSTM1) polymorphism is associated with atopic dermatitis susceptibility in a Korean population. *Int J Immunogenet*. 2011;38(2):145–150.
8. Fu L, et al. Up-regulation of FOXD1 by YAP alleviates senescence and osteoarthritis. *PLoS Biol*. 2019;17(4):e3000201.
9. Pollock RA. Molecular biomarker discovery in psoriatic arthritis. PhD dissertation, University of Toronto. 2016.

Table S6. Gene deletion analysis of the ISG15 kindred [a]

Chr.	Gene GenBank ID	Gene name	Relevant functional implication(s) of gene product	Index case, deletion found (exon)	
				003* Case P1 (m)	004* Case P2 (f)
17	*KRTAP9*	Keratin-associated protein 9	Structure of hair fibers	Exon 3	-
22	*GSTT1*	Glutatione-S-transferase theta 1	Protection against oxidative stress	Exon 1-5 [b]	-
Y	*PCDH11Y*	Protocadherin 11 Y-Linked	Cell-cell recognition in CNS development	Exon 4-8	n/a
Y	*AMELY*	Amelogenin	Mineralization of tooth enamel	Exon 1-3	n/a

[a] WES data were interrogated for homozygous gene (exon) deletions found only in one or both index cases. No deletions were identified in P2.
[b] Common variant (approx. 1/3 of population).

Table S7. List of PCR primers used

**Gene**	Name	Sequence (5´ - 3´)
ISG15	ISG15_F	CAGCGAACTCATCTTTGCCAG
	ISG15_R	GACACCTGGAATTCGTTGCC
HPRT	HPRT_F	GAACGTCTTGCTCGAGATGTG
	HPRT_R	CCAGCAGGTCAGCAAAGAATT
CXCL10	CxCL10_F	CTGCTTTGGGGTTTATCAGA
	CxCL10_R	CCACTGAAAGAATTTGGGC
IFIT1	IFIT1_F	TCAGGCATTTCATCGTCATC
	IFIT1_R	GCAGAACGGCTGCCTAATTT
IFIT2	IFIT2_F	GGAGGGAGAAAACTCCTTGGA
	IFIT2_R	GGCCAGTAGGTTGCACATTGT
OAS1	OAS1_F	TGACTGGCGGCTATAAACC
	OAS1_R	TGGGCTGTGTTGAAATGTGT
MX1	MX1_F	ACAGGACCATCGGAATCTTG
	MX1_R	CCCTTCTTCAGGTGGAACAC
TNF	TNF-α_F	ACCCTCTCTCCCCTGGAAAGGACA
	TNF-α_R	TGAGGAACAAGCACCGCCTGGA
COL1A1	COL1A1_F	AAGCAACCCAAACTGAACCC
	COL1A1_R	TTCAAGCAAGTGGACCAAGC
COL7A1	COL7A1_F	GTGAGGACTGCCCCTGAG
	COL7A1_R	GACTCCACCTTCGAGACCC
COL12A1	COL12A1_F	GTCCCAGGATGAGGTCAAGA
	COL12A1_R	TGGCAAGCTCATTGTAGTCG
COL14A1	COL14A1_F	TCACTTCCTACACGACCACC
	COL14A1_R	GGGTTCCATCTGTGCCAATC
COL15A1	COL15A1_F	CTGGGAGTCCAGAGCTCATC
	COL15A1_R	ATCAAGTGGAGGACCTGGTG
COL5A2	COL5A2_F	CCAGGAGTTCCAGGTTTCAA

	COL5A2_R	CAACTGTTCCTGGGTCACCT
MMP1	MMP1_F	CTGGCCACAACTGCCAAATG
	MMP1_R	CTGTCCCTGAACAGCCCAGTACTTA
TIMP1	TIMP1_F	CCTTCTGCAATTCCGACCTC
	TIMP1_R	GTATCCGCAGACACTCTCCA
TGFB1	TGF-β1_F	TTG AGACTTTTCCGTTGCCG
	TGF-β1_R	CGAGGTCTGGGGAAAAGTCT
ITGA11	ITGA_F	GGTCTGTAAAAGATGTGGTGGAA
	ITGA_R	CTTCTGGAAAGCCTCTGAGC
GPX7	GPX7_F	AACTGGTGTCGCTGGAGAAG
	GPX7_R	AAACTGGTTGCAGGGGAAG
